# Fusing Direct and Indirect Measurements Through Multi‐Fidelity Learning For Accelerated Electrocaloric Materials Discovery

**DOI:** 10.1002/advs.77152

**Published:** 2026-09-03

**Authors:** Bo Wang, Pengfei Dang, Yuan Tian, Zhengwang He, Wei Gu, Lixue Zhang, Turab Lookman, Yumei Zhou, Dezhen Xue

**Affiliations:** ^1^ State Key Laboratory for Mechanical Behavior of Materials Xi'an Jiaotong University Xi'an China; ^2^ College of Materials Science and Engineering Chongqing University Chongqing China

**Keywords:** multi‐fidelity learning, electrocaloric materials, active learning, ferroelectric ceramics, data‐driven materials discovery

## Abstract

The data‐driven discovery of high‐performance electrocaloric (EC) materials is challenged by sparse direct measurements and systematic discrepancies between direct and indirect measurements, resulting in heterogeneous datasets with varying fidelity levels. Here, a co‐kriging‐based multi‐fidelity learning framework is developed to integrate these data sources and construct a robust predictive model for ${\mathrm{BaTiO}}_{3}$‐based ferroelectric ceramics by explicitly modeling cross‐fidelity correlation and discrepancy. Combined with a multi‐objective active learning strategy, the framework enables efficient optimization of low‐temperature EC strength and operational temperature span across the composition–processing space. Guided by this approach, a multi‐element‐doped ${\mathrm{BaTiO}}_{3}$‐based ceramic exhibiting an EC strength of $0.06\times 10^{-6}$ K·m/V at 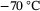 together with a broad operational temperature span of 75 K is identified. Experimental characterization reveals that the enhanced performance originates from a suppressed and diffuse phase transition associated with a relaxor‐like or weakly ordered state, enabling broad temperature stability together with large reversible polarization. These results demonstrate that integrating multi‐fidelity learning with active learning provides an effective strategy for accelerating functional materials discovery under realistic experimental constraints.

## Graphical abstract

A multi‐fidelity framework integrates sparse direct and abundant indirect electrocaloric measurements. Multi‐objective active learning accelerates BaTiO3‐based electrocaloric materials discovery at –70. A diffuse transition enables an electrocaloric strength of 0.06×10^−6^ Km/V at –70℃ with an operational temperature span of 75 K.

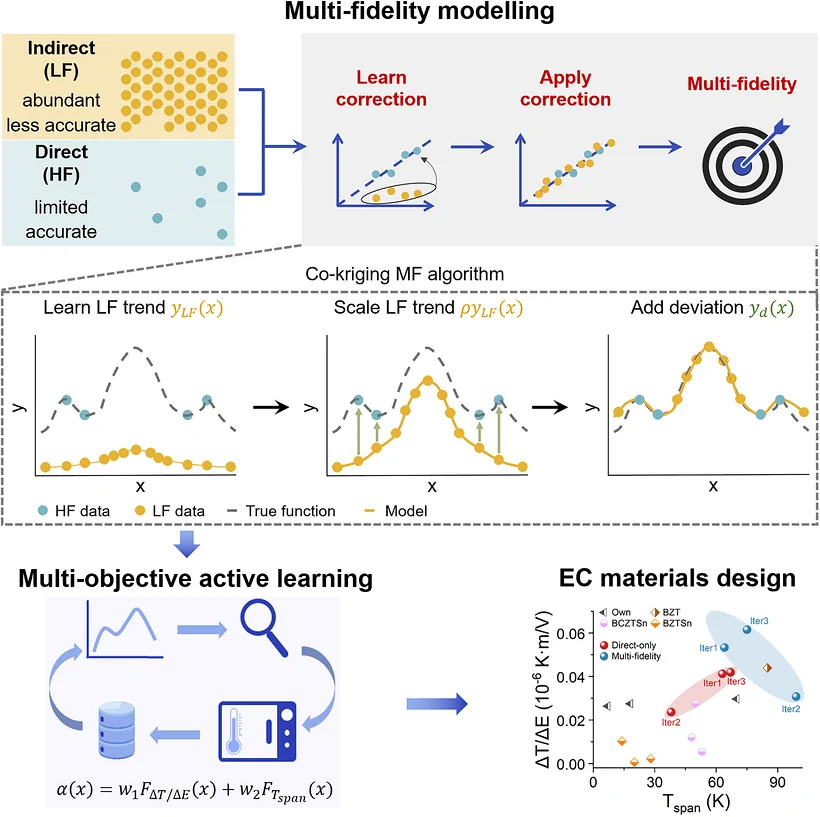

## 1 Introduction

Electrocaloric (EC) cooling has attracted increasing attention as a promising solid‐state refrigeration technology because of its high efficiency and environmental friendliness [1, 2, 3]. For practical ultra‐low‐temperature refrigeration applications, such as biomedical storage and transport near 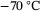, EC materials must simultaneously exhibit large adiabatic temperature change and broad operational temperature span [4, 5, 6]. Large EC response is required to provide sufficient cooling capability [7, 8, 9], while broad operational temperature span enables cascaded refrigeration architectures capable of achieving large temperature lift [10, 11, 12]. However, EC responses are typically strongly coupled to phase transitions and therefore concentrated within narrow temperature spans [13, 14]. Consequently, simultaneously achieving high EC strength and broad operational temperature span remains a major challenge for practical EC refrigeration.

To overcome this limitation, recent studies have employed chemical substitution, defect engineering, and local‐structure regulation to introduce local heterogeneity and diffuse phase transitions, thereby enhancing EC response while broadening the operational temperature span [1, 15, 16]. However, these strategies substantially increase the complexity of the composition–processing space and introduce competing design constraints, making efficient materials optimization difficult using conventional approaches.

Data‐driven methods have therefore emerged as a promising route for accelerating the discovery and optimization of EC materials with targeted properties [17, 18, 19]. Several recent studies have applied machine learning techniques to predict EC behavior and guide materials design [20, 21, 22]. Nevertheless, the effectiveness of these approaches is fundamentally limited by the nature of available EC datasets, which are commonly derived from two distinct measurement methods [23, 24]. Direct measurements, typically performed by monitoring the adiabatic temperature change (ΔT) using temperature or heat‐flux sensors under applied electric fields, provide high‐fidelity EC responses but are experimentally demanding and therefore limited in quantity [25, 26]. In contrast, indirect methods based on thermodynamic Maxwell relations derived from polarization–electric field–temperature measurements are substantially more abundant, but may introduce systematic discrepancies, particularly in materials exhibiting first‐order phase transitions or relaxor behavior [27, 28, 29]. This imbalance between data fidelity and data availability limits reliable data‐driven modeling, especially when both predictive accuracy and broad design‐space coverage are required.

To address these limitations, modeling approaches capable of effectively utilizing heterogeneous datasets while maintaining predictive reliability are required [30]. In this work, a co‐kriging‐based multi‐fidelity learning framework is employed to explicitly model the correlation and discrepancy between direct and indirect EC measurements. It leverages the broad coverage of low‐fidelity (indirect measurement) data while preserving the accuracy of high‐fidelity (direct measurement) information, resulting in improved predictive performance and robustness compared with conventional single‐fidelity approaches. Built upon this surrogate model, a multi‐objective active learning strategy is employed to efficiently explore the composition–processing space of ${\mathrm{BaTiO}}_{3}$‐based bulk ferroelectric ceramics. Using this integrated framework, a multi‐element‐doped ${\mathrm{BaTiO}}_{3}$‐based ceramic, $\left({\mathrm{Ba}}_{0.93}{\mathrm{Ca}}_{0.06}{\mathrm{Sr}}_{0.01}\right)\left({\mathrm{Ti}}_{0.75}{\mathrm{Zr}}_{0.19}{\mathrm{Sn}}_{0.01}{\mathrm{Hf}}_{0.05}\right)O_{3}$, is identified, exhibiting an EC strength of $0.06\times 10^{-6}$ K·m/V at 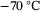 together with a broad operational temperature span of 75 K. Overall, the combination of multi‐fidelity learning and active learning provides an effective framework for overcoming data limitations and accelerating the discovery of functional ceramic materials.

## 2 Results and Discussion

### 2.1 Design Strategy

#### 2.1.1 Multi‐Fidelity Modeling of Electrocaloric Data

**FIGURE 1:**
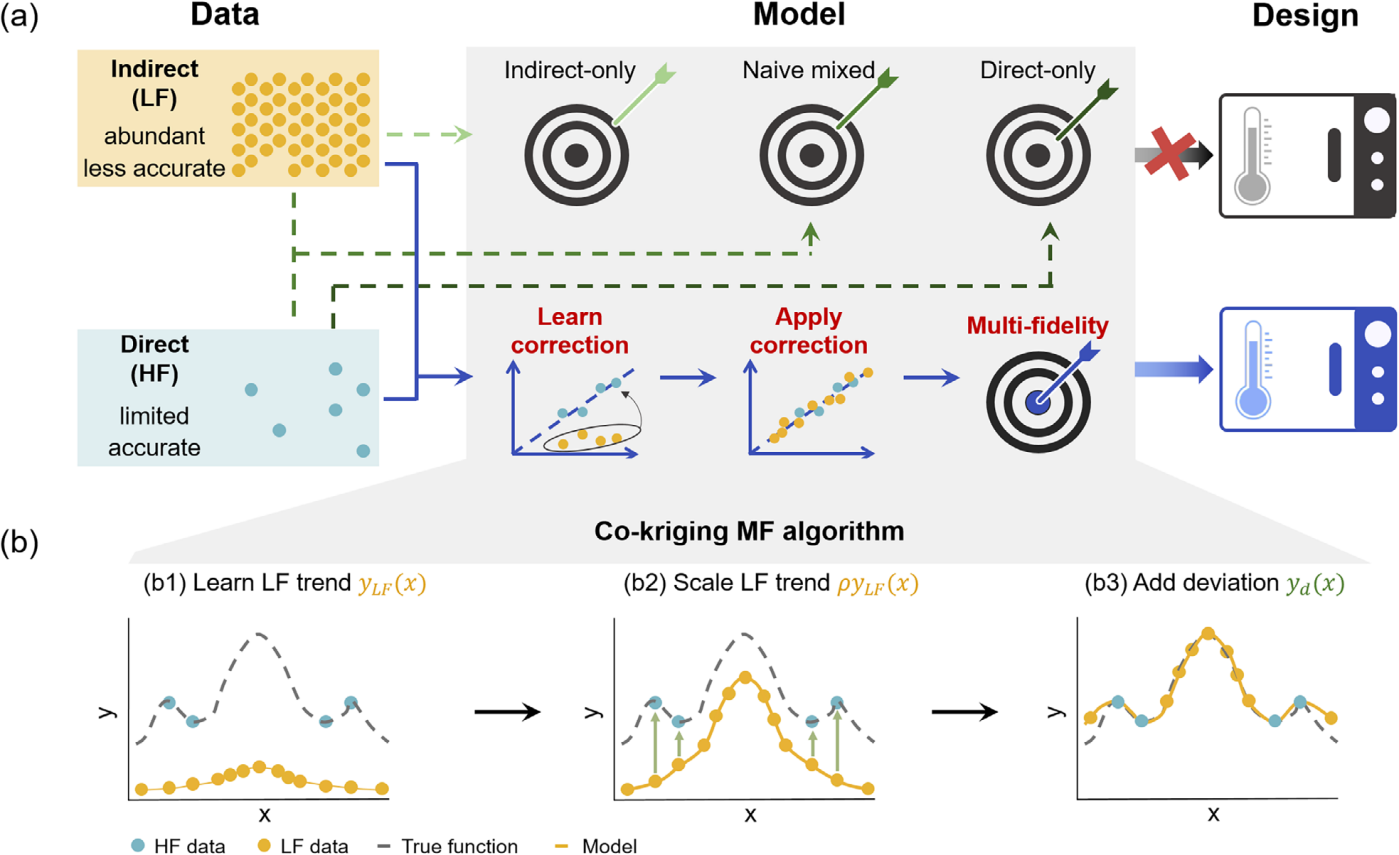
Schematic illustration of the multi‐fidelity learning framework for electrocaloric (EC) materials design. (a) Comparison between conventional modeling strategies and the proposed multi‐fidelity approach. Indirect‐only, Direct‐only, and Naive mixed models suffer from limited data coverage or systematic bias, whereas the multi‐fidelity framework explicitly learns the relationship between low‐fidelity (LF) and high‐fidelity (HF) data to improve predictive reliability and design efficiency. (b) Formulation of the co‐kriging‐based multi‐fidelity model. The LF response is first globally scaled and subsequently corrected using a discrepancy function to reconstruct the HF response according to $y_{HF}\left(x\right)=\rho \;y_{LF}\left(x\right)+y_{d}\left(x\right)$.

As shown in Figure 1a, EC datasets commonly consist of measurements with different fidelity levels. Direct EC measurements provide high‐fidelity (HF) responses but are limited in quantity because they require specialized measurement systems and quasi‐adiabatic conditions, whereas indirect measurements derived from thermodynamic relations provide substantially more abundant low‐fidelity (LF) data with broader coverage of the composition–processing–testing space. This imbalance between data fidelity and data availability makes reliable modeling challenging. Models trained solely on either dataset are inherently limited, while naive combination of HF and LF data may bias predictions toward low‐fidelity responses because of the dominance of LF data. To address this issue, a multi‐fidelity learning strategy is adopted to leverage the complementary strengths of both data sources. Rather than directly merging the datasets, the approach explicitly models the correlation and discrepancy between LF and HF measurements, enabling low‐fidelity information to be systematically corrected using high‐fidelity observations.

This conceptual framework is formalized using a co‐kriging‐based multi‐fidelity model, as illustrated in Figure 1b. The approach is formulated as a two‐step correction process. First, the low‐fidelity (LF) response is globally scaled to match the overall trend of the high‐fidelity (HF) data,

(1) $${\tilde{y}}_{LF}\left(x\right)=\rho \;y_{LF}\left(x\right),$$

where ρ is a scaling coefficient relating the two fidelity levels. Second, a discrepancy function is introduced to account for systematic deviations between the scaled LF prediction and the HF response,

(2) $$y_{HF}\left(x\right)={\tilde{y}}_{LF}\left(x\right)+y_{d}\left(x\right),$$

where $y_{d}\left(x\right)$ represents the residual discrepancy between the two datasets. Combining these two steps yields the co‐kriging relation [31, 32],

(3) $$y_{HF}\left(x\right)=\rho \;y_{LF}\left(x\right)+y_{d}\left(x\right).$$

This formulation enables the model to capture global trends from abundant LF data while correcting local discrepancies using more reliable HF observations.

Both the low‐fidelity response $y_{LF}\left(x\right)$ and the discrepancy function $y_{d}\left(x\right)$ are modeled using Gaussian process (GP) regressors. For a given material feature vector x, the EC response is treated as a Gaussian random field characterized by a mean function and a covariance kernel. The model is trained using the low‐ and high‐fidelity datasets, $\left(X_{LF},y_{LF}\right)$ and $\left(X_{HF},y_{HF}\right)$, and provides predictive means together with associated uncertainties for new inputs. Model parameters, including the scaling coefficient ρ and kernel hyperparameters, are determined through maximum likelihood estimation. Detailed formulations are provided in the Supporting Information. For fair comparison, the Gaussian process regressor is also adopted for the single‐fidelity models: Indirect‐only (trained using low‐fidelity data), Direct‐only (trained using high‐fidelity data), and Naive mixed (trained using directly combined low‐ and high‐fidelity data).

In this work, the target response y is the EC strength, defined as ΔT/ΔE [13, 33]. The material feature vector x includes composition descriptors, processing parameters, and testing conditions, including temperature T and electric‐field change ΔE. Detailed definitions of these descriptors are provided in the Supporting Information.

To ensure rigorous evaluation of model generalization, training–validation splits were performed in the composition–processing space rather than at the individual data‐point level. Accordingly, all measurements associated with the same material were assigned to the same subset, preventing data obtained under different testing conditions from simultaneously appearing in both training and validation datasets. This strategy enables model performance to be evaluated on previously unseen materials.

#### 2.1.2 Multi‐Objective Active Learning For Materials Optimization

To efficiently explore the composition–processing space, a multi‐objective active learning strategy is constructed based on the multi‐fidelity surrogate model. Two objectives are considered simultaneously: EC strength at 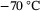 and operational temperature span $T_{span}$, defined as the temperature range over which ΔT exceeds 80% of its maximum value [20, 34]. These objectives are inherently competing because materials with sharp phase transitions typically exhibit high peak EC response but narrow operating spans.

The multi‐fidelity model provides predictive means and uncertainties across the design space. At each iteration, candidate points are selected by maximizing a multi‐objective acquisition function,

(4) $$\alpha \left(x\right)=w_{1}F_{\Delta T/\Delta E}\left(x\right)+w_{2}F_{T_{span}}\left(x\right),$$

where $F_{\Delta T/\Delta E}$ and $F_{T_{span}}$ denote the acquisition functions for EC strength and temperature span, respectively, and $w_{1}$ and $w_{2}$ are weighting factors. Because EC strength and operational temperature span have different numerical scales, the corresponding acquisition functions were scaled to comparable ranges before combination. Equal contribution from the two objectives was adopted by assigning balanced weights. Both acquisition functions are evaluated using the expected improvement (EI) criterion, which balances exploration and exploitation through predictive uncertainty (see Supporting Information).

The active learning process is implemented in a closed loop. Selected candidates are synthesized and characterized using direct EC measurements, and the resulting high‐fidelity data are incorporated to update the surrogate model. Through iterative refinement, the framework efficiently guides exploration toward materials exhibiting strong EC response at low temperature (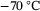) together with broad operational temperature span.

### 2.2 Characteristics of Direct and Indirect EC Datasets

**FIGURE 2:**
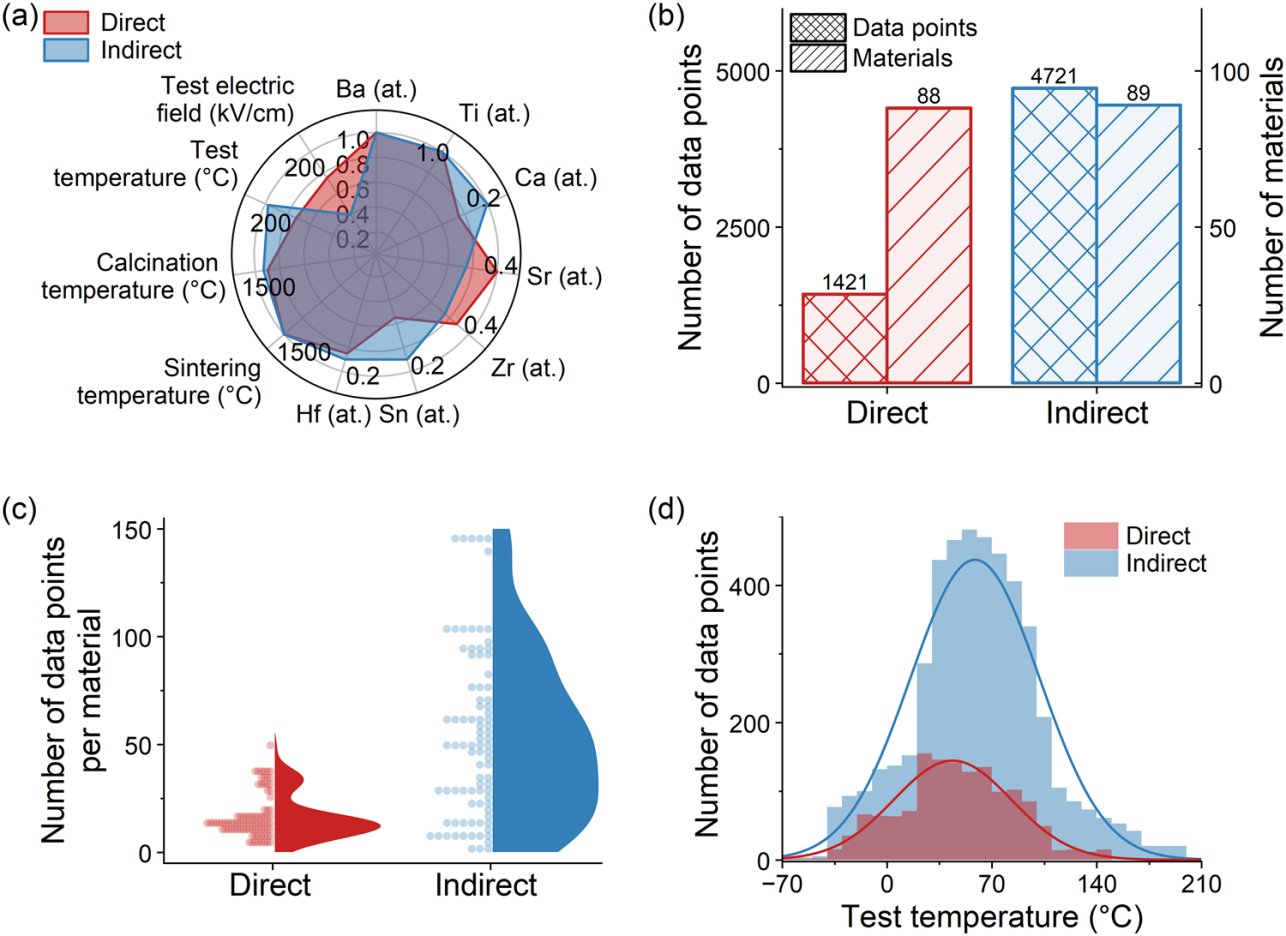
Overview of the electrocaloric (EC) dataset, highlighting the heterogeneity between high‐fidelity (direct) and low‐fidelity (indirect) data. (a) Feature space coverage, including composition, processing parameters, and testing conditions. (b) Total number of data points and unique materials, showing that indirect data are substantially more abundant while spanning a comparable number of material systems. (c) Distribution of data points per material, indicating significantly denser sampling in the indirect dataset. (d) Distribution of test temperature, illustrating broader temperature coverage for indirect measurements.

The characteristics of the EC dataset used in this work are summarized in Figure 2. The dataset was compiled from the literature on ${\mathrm{BaTiO}}_{3}$‐based EC ceramics, combining direct EC measurements with indirect data derived from thermodynamic Maxwell relations. As shown in Figure 2a, the two data sources exhibit distinct coverage in the feature space, including composition, processing parameters, and testing conditions.

As shown in Figure 2b, the indirect dataset is substantially larger than the direct dataset, containing 4721 data points compared to 1421 for direct measurements, while both datasets span a comparable number of distinct materials. In this work, a “distinct material” is defined by its nominal composition and processing conditions. A “data point” corresponds to one electrocaloric measurement obtained for a given distinct material under specific testing conditions, including temperature and electric‐field change. Therefore, one distinct material may correspond to multiple data points when characterized under different measurement conditions. This indicates that the primary difference between the two datasets arises from sampling density and measurement modality rather than the range of materials covered. Indirect data further provide significantly denser sampling for each material system, whereas direct measurements remain comparatively sparse, as shown in Figure 2c. Furthermore, Figure 2d reveals broader temperature coverage for indirect data, while direct measurements are distributed over a more limited range.

Taken together, these observations indicate pronounced data imbalance and distribution mismatch across fidelity levels. The dominance of low‐fidelity data can bias conventional single‐fidelity models and reduce predictive reliability for high‐fidelity responses, thereby motivating the use of a multi‐fidelity framework capable of explicitly modeling correlations and discrepancies between fidelity levels.

### 2.3 Multi‐Fidelity Modeling of EC Responses

**FIGURE 3:**
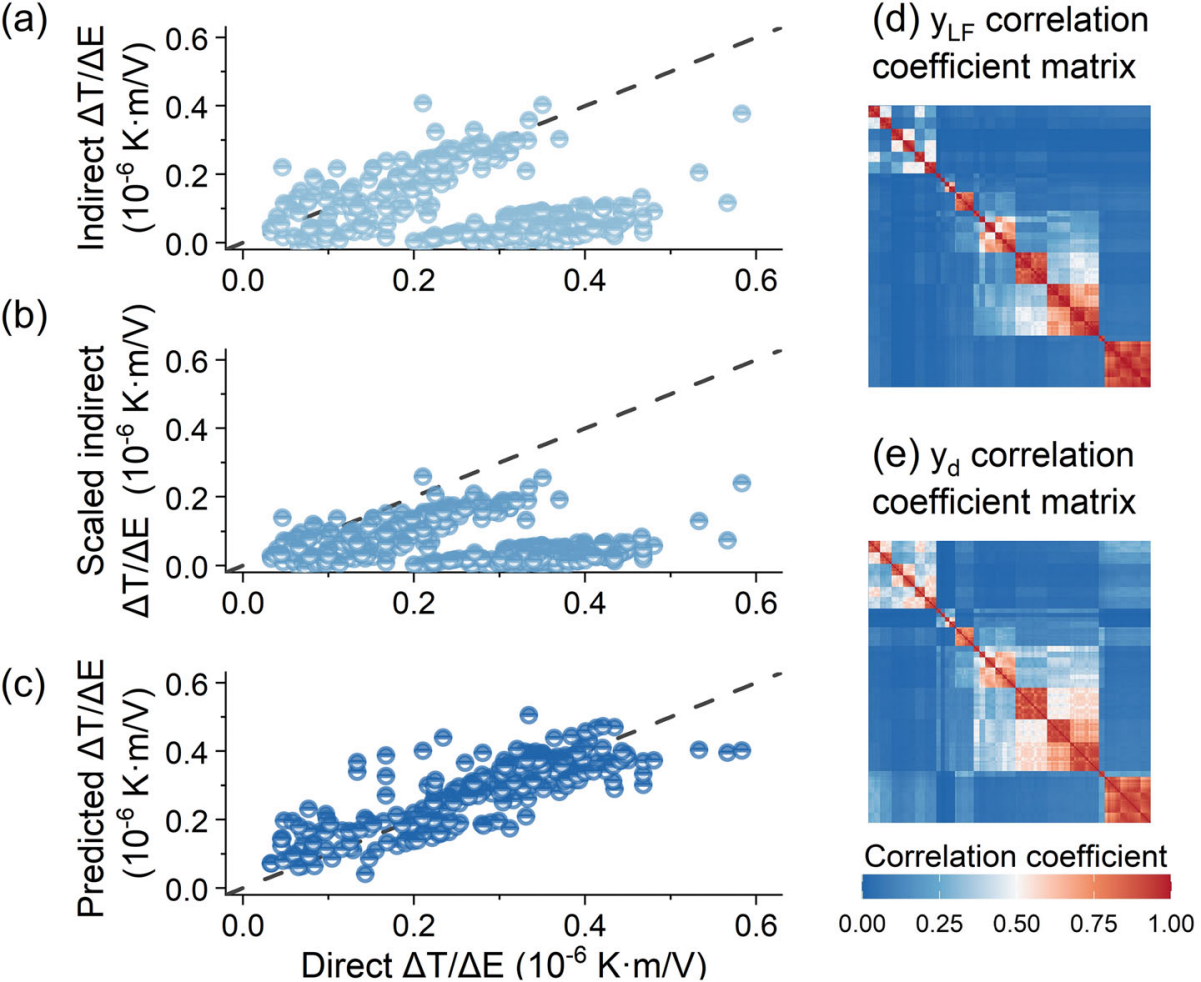
Evaluation of the multi‐fidelity modeling framework using co‐located electrocaloric (EC) strength data (i.e., identical composition, processing parameters, and testing conditions across fidelity levels). (a) Direct (high‐fidelity) vs. indirect (low‐fidelity) EC strength, showing significant scatter and deviation from the one‐to‐one line, indicative of systematic discrepancies between the two data sources. (b) Indirect EC strength after global scaling using the co‐kriging correlation coefficient, reducing overall deviation while retaining noticeable inconsistencies. (c) Predicted EC strength from the multi‐fidelity model vs. direct measurements, demonstrating substantially improved agreement with high‐fidelity responses. (d) Correlation matrix of the low‐fidelity Gaussian process component, revealing coherent global similarity patterns across the design space. (e) Correlation matrix of the discrepancy component, showing localized variations associated with systematic differences between fidelity levels.

The effectiveness of the multi‐fidelity framework is evaluated using co‐located EC data, defined as measurements obtained under identical composition, processing parameters, and testing conditions for both direct and indirect methods, as shown in Figure 3. Figure 3a–c illustrates the corresponding two‐step correction process described in Figure 1b.

As shown in Figure 3a, indirect EC values exhibit weak correlation with direct measurements and deviate substantially from the one‐to‐one line (y=x), indicating systematic discrepancies between the two data sources. Applying global scaling using the co‐kriging coefficient partially improves the agreement by reducing systematic deviation (Figure 3b); however, noticeable discrepancies remain, particularly at higher EC values. Then, the multi‐fidelity model, which incorporates both global scaling and local discrepancy correction, produces predictions closely aligned with direct measurements (Figure 3c), indicating that the model effectively captures both global trends and local variations in the high‐fidelity response.

Further insight into the model behavior is provided by the correlation (normalized Gaussian process covariance kernel) matrices shown in Figure 3d,e. The low‐fidelity component exhibits a coherent block structure associated with global similarity patterns, whereas the discrepancy component shows more localized variations corresponding to systematic differences between scaled indirect and direct measurements. Together, these results demonstrate that the multi‐fidelity framework effectively combines global trends and local corrections to reconstruct high‐fidelity EC responses.

To facilitate co‐kriging, which requires co‐located data pairs, an auxiliary data‐alignment procedure was introduced to increase the number of paired observations. A predictive model trained on indirect data was used to estimate corresponding values at non‐co‐located high‐fidelity positions, increasing the number of paired data from 328 to 1421 (Figure S4). This procedure was used solely for data alignment and was not involved in subsequent model validation or performance evaluation.

**FIGURE 4:**
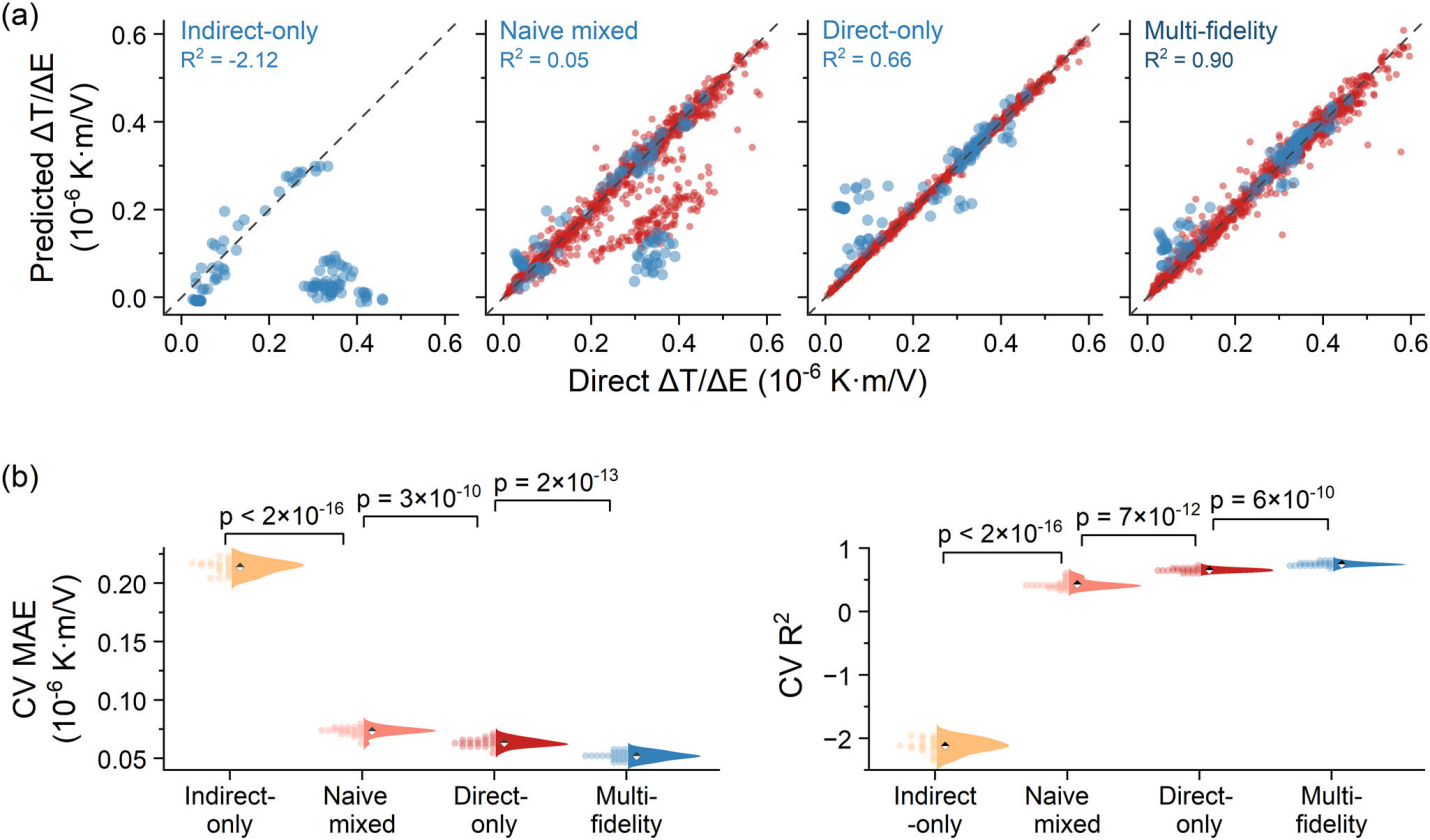
Comparison of predictive performance for different modeling approaches based on cross‐validation. (a) Representative results from one fold of 10‐fold cross‐validation, showing predicted vs. directly measured electrocaloric (EC) strength. Blue and red symbols denote validation and training data, respectively. From left to right: Indirect‐only, Naive mixed, Direct‐only, and Multi‐fidelity (MF) models. The coefficient of determination ($R^{2}$) is indicated for each case. (b) Statistical results from 20 repetitions of 10‐fold cross‐validation. Distributions of mean absolute error (MAE, left) and $R^{2}$ (right) are shown. The MF model consistently achieves lower prediction error and higher $R^{2}$ compared with the Indirect‐only, Naive mixed, and Direct‐only models, with statistically significant improvement over the Direct‐only model based on the p‐values.

The predictive performance of the model is further evaluated through cross‐validation, as summarized in Figure 4. Representative results from one fold of the 10‐fold cross‐validation are shown in Figure 4a, where predicted EC strengths are compared with direct measurements.

The indirect‐only model exhibits extremely poor performance, with a negative coefficient of determination ($R^{2}=-2.12$), indicating that predictions are worse than a constant estimator. The naively mixed model shows only marginal improvement ($R^{2}=0.05$), suggesting that the dominance of low‐fidelity data biases the learning process. The direct‐only model achieves moderate accuracy ($R^{2}=0.66$), reflecting the reliability of high‐fidelity data but also the limitation imposed by data sparsity.

In contrast, the multi‐fidelity model shows substantial improvement, with predictions closely aligned along the one‐to‐one line and a high coefficient of determination ($R^{2}=0.90$). This result confirms that incorporating low‐fidelity information through explicit discrepancy correction substantially improves predictive performance.

Statistical results from 20 repetitions of 10‐fold cross‐validation, shown in Figure 4b, further confirm the robustness of the approach. The multi‐fidelity model consistently achieves the lowest mean absolute error and the highest $R^{2}$ among all methods, with statistically significant improvement over the direct‐only model, as indicated by the reported p‐values. Overall, these results demonstrate that the proposed multi‐fidelity framework improves predictive accuracy while maintaining robust performance under different training‐validation partitions.

### 2.4 Generalization and Impact of Low‐Fidelity Data

**FIGURE 5:**
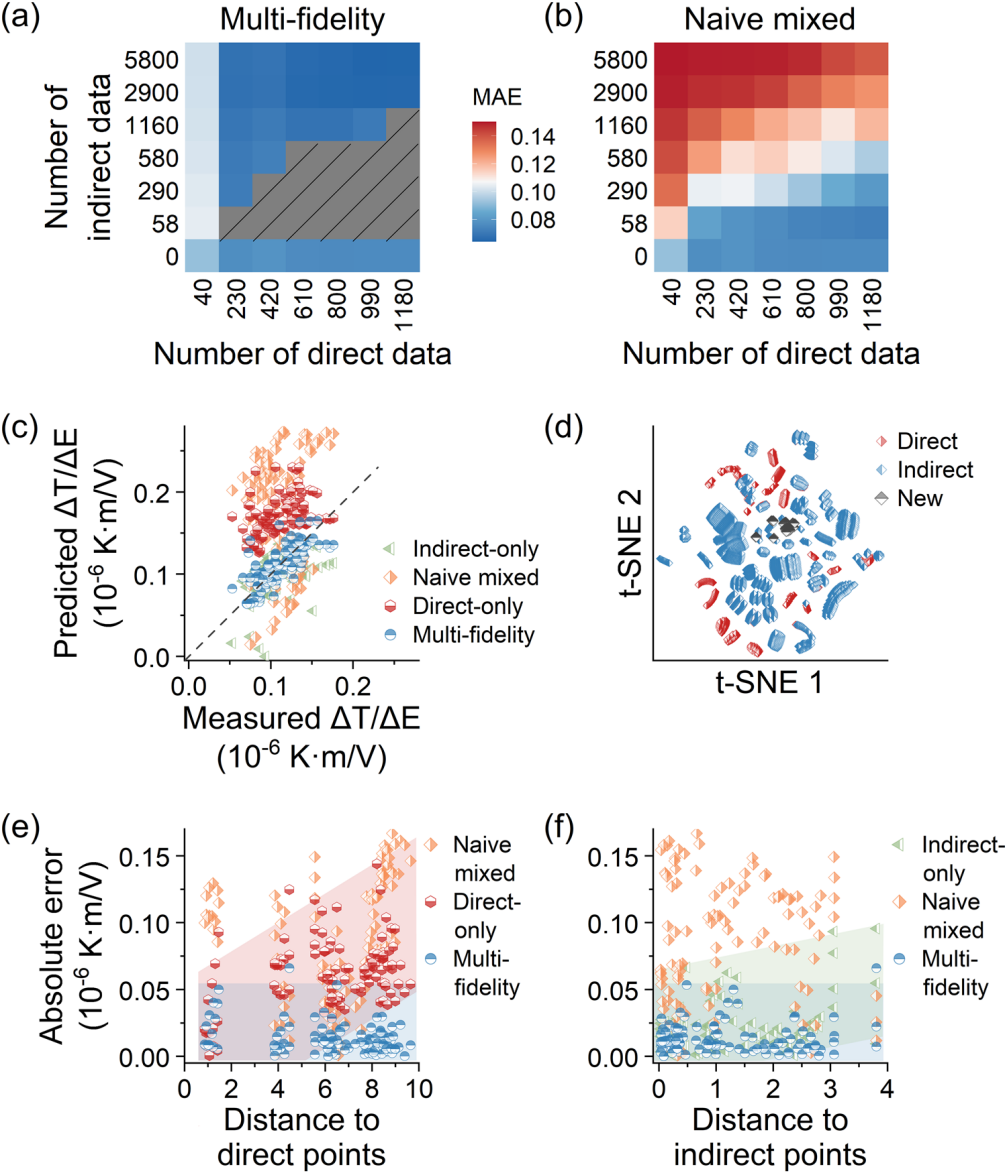
Evaluation of the multi‐fidelity (MF) framework in terms of predictive performance and generalization. (a) Mean absolute error (MAE) of the MF model as a function of the number of direct and indirect data points, showing systematic error reduction with increasing indirect data. (b) MAE heatmap for the Naive mixed model under the same data configurations, showing performance degradation with increasing indirect data. (c) Predicted vs. measured electrocaloric (EC) strength for newly synthesized samples using different models. (d) t‐SNE visualization of direct, indirect, and newly synthesized data in feature space. (e–f) Absolute prediction error as a function of distance to the nearest direct (e) and indirect (f) data points. The MF model exhibits lower prediction error and weaker dependence on data distance compared with single‐fidelity models.

The impact of low‐fidelity data on predictive performance and generalization is further evaluated in Figure 5. A key question is whether increasing the amount of indirect data improves prediction accuracy when combined with limited high‐fidelity observations. As shown in Figure 5a, the prediction error of the multi‐fidelity model decreases systematically with increasing amounts of indirect data, except in the extreme low‐data regime where only 40 direct data points are available. In contrast, the Naive mixed model shows no comparable improvement and even deteriorates with increasing indirect data (Figure 5b). The prediction errors are evaluated on a 10% direct data validation set. The trends are statistically robust, as each mean absolute error (MAE) value represents the average over 25 independent validation runs generated from repeated random training‐validation splits and training‐data resampling. Together, these results demonstrate that low‐fidelity data improve predictive performance only when their systematic discrepancies from high‐fidelity data are explicitly modeled.

Because co‐kriging requires a nested multi‐fidelity structure ($X_{HF}\subset X_{LF}$), only data configurations with the number of indirect data no smaller than that of direct data are feasible. The infeasible configurations are therefore omitted and indicated by the grey hatched region in Figure 5a.

The predictive capability of the model is further assessed using newly synthesized samples, as shown in Figure 5c. To ensure physically meaningful compositions, the candidate space was constrained to non‐toxic dopant elements and composition ranges satisfying the tolerance‐factor criterion for suppressing secondary phase formation. Twelve compositions were randomly selected from the resulting ${\mathrm{BaTiO}}_{3}$‐based design space and experimentally synthesized (see Table S2). Random selection was adopted for independent model validation to avoid model‐dependent selection bias, because different surrogate models may identify different regions as uncertain or promising. Although this strategy does not specifically probe high‐uncertainty regions, it provides a model‐independent external validation set for comparing predictive performance among different approaches. In contrast, uncertainty was explicitly considered during active‐learning‐based materials optimization through the expected‐improvement acquisition function. The multi‐fidelity model shows the closest agreement with experimental measurements among all models, demonstrating substantially improved generalization beyond the training dataset.

To elucidate the origin of this improvement, the feature‐space distribution is analyzed using t‐SNE, as shown in Figure 5d. The newly synthesized samples are distributed closer to the indirect‐data domain than to the direct‐data domain, indicating that indirect measurements provide substantially broader coverage of the relevant design space. This broader coverage is expected to improve prediction reliability in previously unexplored regions.

This interpretation is further supported by the relationship between prediction error and distance to existing data points, as shown in Figure 5e,f. Both the Direct‐only and Indirect‐only models exhibit increasing prediction error as the distance from existing data increases, indicating strong dependence on nearby training samples. In contrast, the multi‐fidelity model remains substantially less sensitive to the distance from either direct or indirect data, while consistently maintaining lower prediction error. These results indicate that the multi‐fidelity framework captures the underlying global trends of the EC response rather than relying predominantly on local interpolation.

Overall, these results demonstrate that the improved generalization capability of the multi‐fidelity framework originates from its ability to leverage the broader design‐space coverage provided by low‐fidelity data while correcting their systematic discrepancies through high‐fidelity observations. This combination enables robust prediction in previously unexplored regions of the materials space.

### 2.5 Evaluation of Active Learning Efficiency Based on the Multi‐Fidelity Model

**FIGURE 6:**
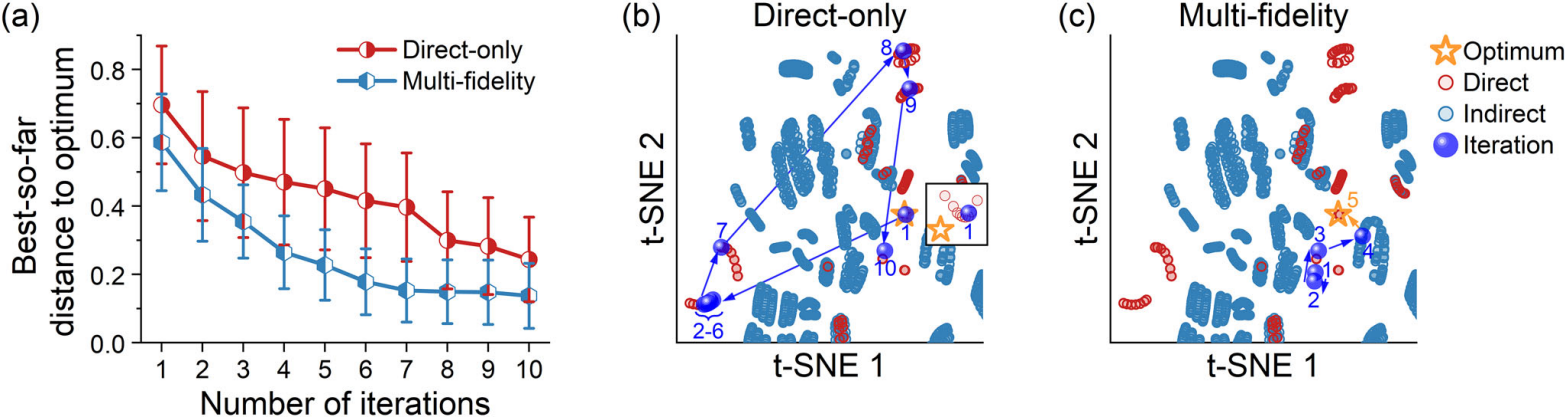
Comparison of active learning efficiency for the multi‐fidelity (MF) and Direct‐only models under a simulated search scenario. The dataset is randomly divided into training and virtual candidate sets, with the optimal target point excluded from the initial training data. (a) Distance between the best candidate identified at each iteration and the target point, showing faster convergence of the MF model toward the optimal region. (b) t‐SNE visualization of the sampling trajectory for the Direct‐only model (single random split), showing exploration primarily confined to regions densely populated by direct data. (c) Corresponding sampling trajectory for the MF model, showing broader exploration enabled by indirect‐data coverage.

The effectiveness of the multi‐fidelity framework in improving search efficiency is evaluated using a simulated active learning scenario, as shown in Figure 6. The dataset is randomly divided into a training set and a virtual candidate set, with the optimal target point (i.e., the material exhibiting the maximum ΔT/ΔE) intentionally excluded from the initial training data. At each iteration, candidate points are selected from the virtual set according to the acquisition function, simulating an iterative materials optimization process.

As shown in Figure 6a, the multi‐fidelity model converges toward the optimal region substantially faster than the Direct‐only model. The distance between the best identified candidate and the target point is evaluated in the normalized composition–processing feature space. To reduce statistical fluctuations associated with dataset partitioning, the training and virtual sets were randomly divided 20 times, and the average values together with the corresponding standard errors are shown in Figure 6a. The multi‐fidelity model exhibits a significantly faster reduction in distance to the target point, indicating more efficient exploration of the design space.

The difference in search behavior is further illustrated by the t‐SNE representations of one representative random split in Figure 6b,c. The Direct‐only model primarily samples regions densely populated by high‐fidelity data, resulting in slower convergence and limited exploration of under‐sampled regions. In contrast, the multi‐fidelity model explores regions that are sparsely covered by direct measurements but supported by indirect data, enabling identification of promising candidates beyond the immediate vicinity of the initial training set and more rapid convergence toward the optimal region.

These results demonstrate that incorporating low‐fidelity information improves the ability of active learning to explore under‐sampled regions of the design space. By leveraging the broader coverage provided by indirect data while maintaining high‐fidelity accuracy through discrepancy correction, the multi‐fidelity framework enables more efficient search and accelerated convergence toward optimal candidates.

### 2.6 Experimental Validation and Performance of Discovered Materials

The proposed multi‐fidelity and multi‐objective active learning framework was further evaluated through experimental discovery of optimized EC materials. The active learning process followed the strategy described in Subsection 2.1.2 using a candidate space constrained by non‐toxic dopant elements and tolerance‐factor requirements for suppressing secondary phases. The selected compositions and processing parameters at each iteration are summarized in Table S3.

**FIGURE 7:**
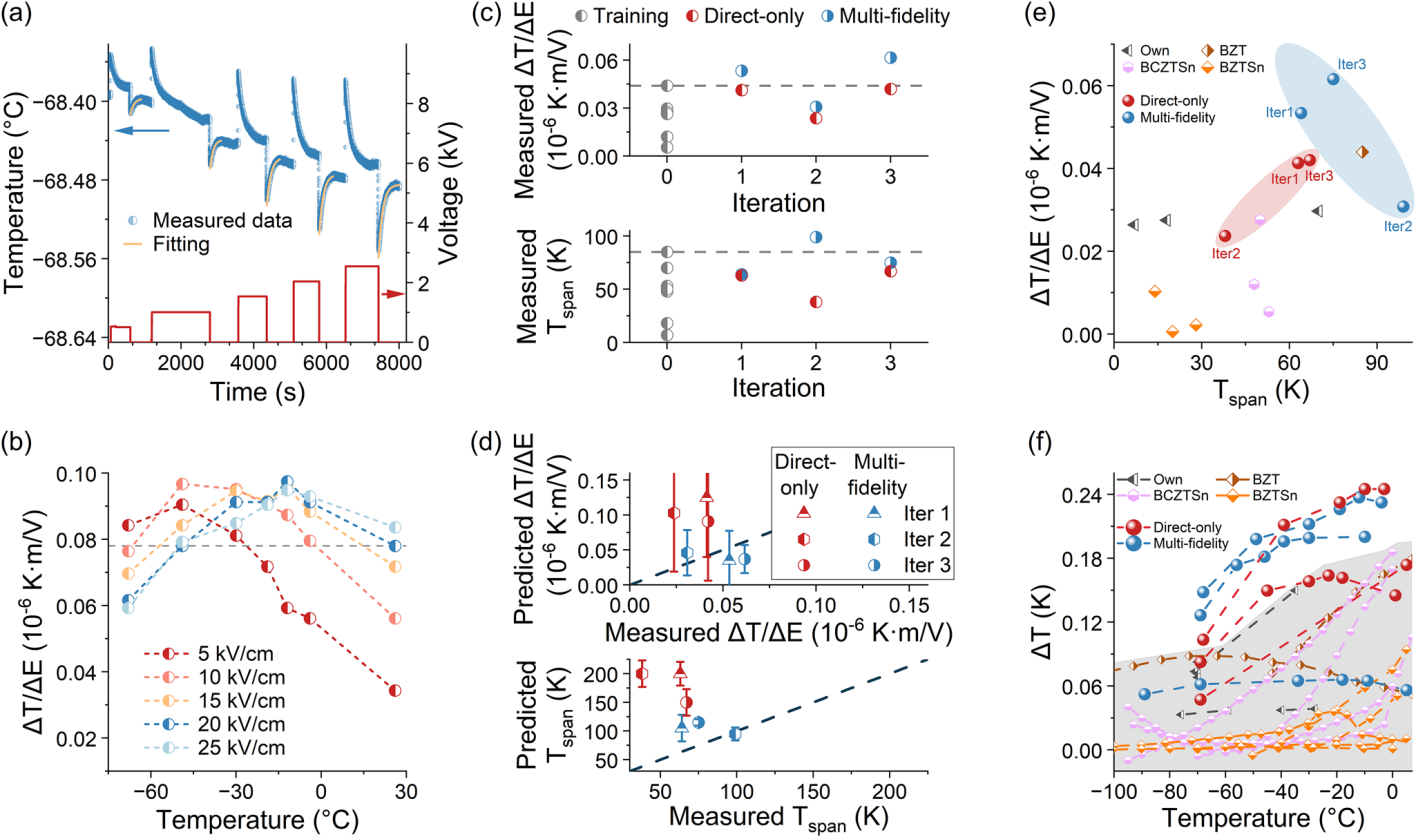
Experimental validation of electrocaloric (EC) materials discovered using the proposed multi‐fidelity active‐learning framework. (a) Direct EC measurement of the final optimized material under step electric‐field pulses at approximately 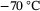. The intrinsic ΔT is obtained by fitting the thermal relaxation behavior and extrapolating to the moment when the electric field is removed. (b) EC strength as a function of temperature under different applied electric fields, showing strong response and a broad operational temperature span. (c) Evolution of measured EC strength at 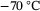 and operational temperature span ($T_{span}$) during active‐learning iterations. (d) Comparison between predicted and experimentally measured EC performance, showing improved predictive accuracy for the multi‐fidelity model. (e) EC strength vs. operational temperature span compared with literature‐reported lead‐free bulk materials measured near 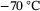. (f) EC temperature change (ΔT) as a function of temperature, demonstrating promising low‐temperature EC performance relative to literature reports.

The EC response was evaluated using direct measurements, as shown in Figure 7a. Step electric‐field pulses (5–25 kV/cm) were applied under quasi‐adiabatic conditions. Because thermal relaxation causes the measured temperature minimum to occur after removal of the electric field, the instantaneous temperature change was obtained by fitting the relaxation behavior and extrapolating to the moment of field removal [35, 36]. A heat‐capacity correction factor was applied to account for the thermal mass of the measurement system (see Supporting Information).

After the third active‐learning iteration, the multi‐fidelity framework identified a multi‐element‐doped ${\mathrm{BaTiO}}_{3}$‐based ceramic, $\left({\mathrm{Ba}}_{0.93}{\mathrm{Ca}}_{0.06}{\mathrm{Sr}}_{0.01}\right)\left({\mathrm{Ti}}_{0.75}{\mathrm{Zr}}_{0.19}{\mathrm{Sn}}_{0.01}{\mathrm{Hf}}_{0.05}\right)O_{3}$, exhibiting an EC strength of $0.06\times 10^{-6}$ K·m/V at 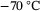 together with a broad operational temperature span of 75 K. As shown in Figure 7b, the optimized material exhibits pronounced EC response over a broad temperature range under different electric fields. Notably, strong EC performance is retained near 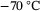, consistent with the targeted application scenario. To evaluate the experimental uncertainty of the reported EC strength, five repeated direct EC measurements were performed for the identified composition near 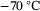. The measured EC strength was $0.062\pm 0.002\times 10^{-6}$ K·m/V, indicating good repeatability of the direct EC measurement (see Supporting Information).

The evolution of model predictions and experimental measurements during active learning is summarized in Figure 7c,d. The multi‐fidelity framework rapidly identified improved candidates within the early active‐learning iterations, achieving enhanced ΔT/ΔE after the first iteration and favorable $T_{span}$ within two iterations. In contrast, the Direct‐only model did not identify candidates with substantially improved performance relative to the initial training set. Moreover, predictions from the multi‐fidelity model show good agreement with experimental measurements, whereas the Direct‐only model systematically overestimates material performance. These results demonstrate that incorporating low‐fidelity information improves both the efficiency and reliability of surrogate‐guided materials optimization. It should be noted that the purpose of the present active‐learning experiment was to demonstrate data‐efficient materials improvement rather than to establish global convergence of the optimization process. The active‐learning loop was terminated after three iterations because the identified material achieved simultaneous improvement in EC strength and operational temperature span, while further iterations would require substantial experimental resources due to the time‐consuming synthesis and low‐temperature direct EC measurements. Additional iterations may further improve material performance and enable more systematic convergence analysis in future studies.

Finally, Figure 7e,f compares the discovered materials with literature‐reported EC systems. Only lead‐free bulk materials measured near 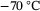 were included for comparison. Because reports in this temperature regime remain limited, both direct and indirect measurement results were considered. The compared EC values were obtained within a narrow temperature range of −73 to 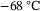. BCZTSn and BZTSn were measured at 8 and 8.7 kV/cm, respectively, whereas the remaining literature‐reported materials were measured at 20 kV/cm. These differences in measurement conditions should be considered when interpreting the comparison. A broader comparison including thin films, single crystals, and lead‐containing systems is provided in the Figure S6 for completeness. Abbreviations used for literature materials are summarized in Table S4. The optimized materials exhibit a favorable combination of EC strength and operational temperature span together with promising EC temperature change (ΔT) under subzero conditions. These results confirm that combining multi‐fidelity learning with multi‐objective active learning enables efficient discovery of EC materials under realistic subzero refrigeration requirements.

### 2.7 Structural Origin of Enhanced Electrocaloric Performance

**FIGURE 8:**
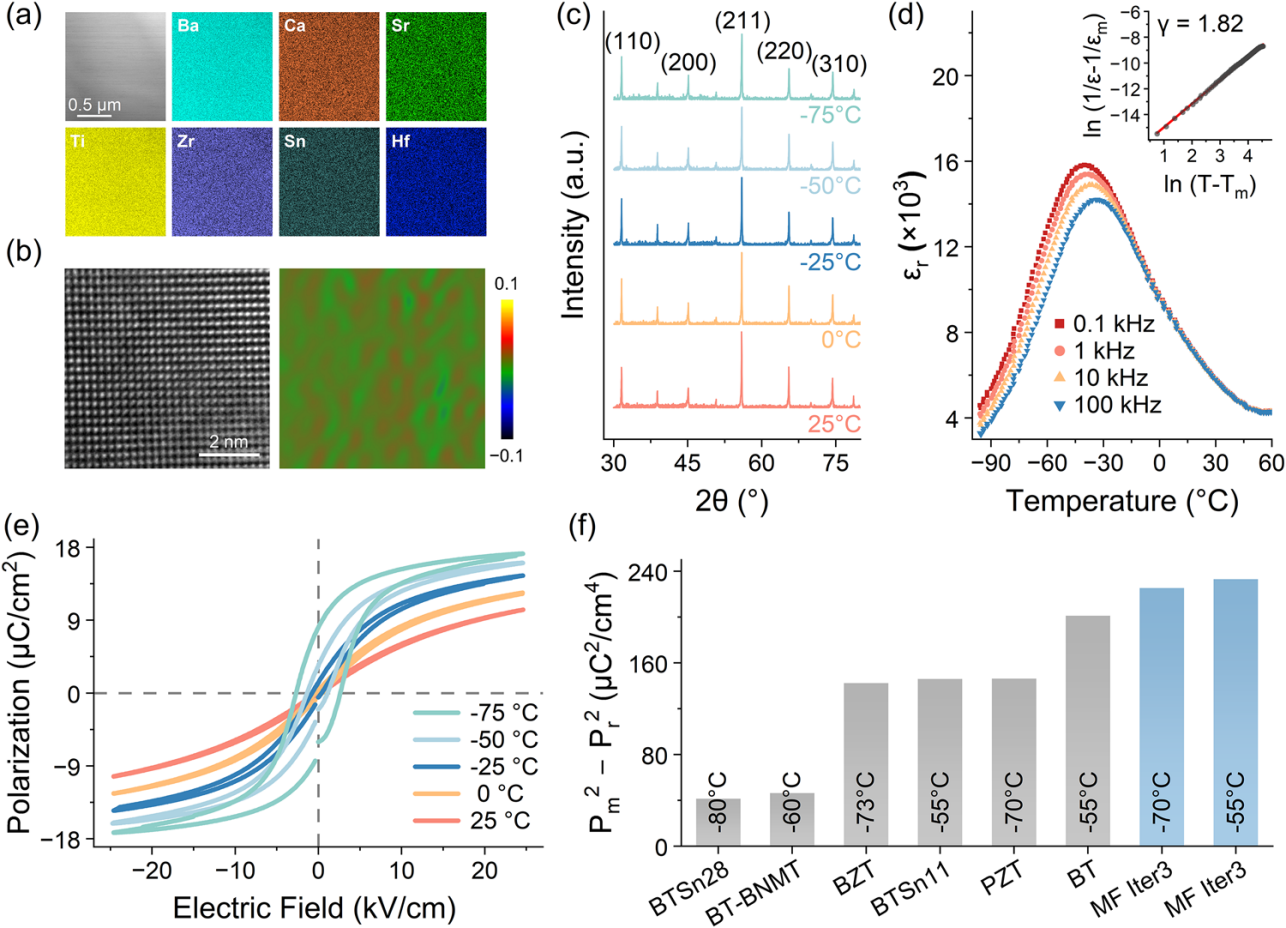
Structural and functional characterization of the optimized electrocaloric material. (a) EDS elemental mapping showing homogeneous distribution of multiple dopant elements. (b) High‐resolution TEM and strain analysis showing nanoscale structural distortions without well‐defined ferroelectric domains. (c) Temperature‐dependent XRD patterns indicating a stable pseudocubic structure without observable symmetry change or peak splitting. (d) Dielectric permittivity as a function of temperature at different frequencies, showing a broad frequency‐dependent peak consistent with relaxor‐like behavior; inset shows the modified Curie–Weiss fitting. (e) Polarization–electric field (P–E) hysteresis loops measured at different temperatures, showing slim loops with low remanent polarization. (f) Comparison of polarization characteristics with literature‐reported materials, highlighting the large field‐induced polarization and low remanence of the present material. All $P_{m}$ values were measured at E=20 kV/cm.

To elucidate the physical origin of the enhanced EC performance, compositional, structural, and functional characterizations were performed, as summarized in Figure 8. The results consistently indicate a suppressed and diffuse phase transition associated with a relaxor‐like state, enabling both broad operational temperature span and strong EC response at low temperature.

Energy‐dispersive spectroscopy (EDS) mapping, shown in Figure 8a, confirms homogeneous distribution of multiple dopant elements throughout the material. Such compositional disorder introduces local structural heterogeneity that suppresses long‐range ferroelectric ordering and promotes relaxor‐like behavior.

Consistent with this interpretation, high‐resolution TEM and strain analysis (Figure 8b) reveal nanoscale structural distortions without well‐defined ferroelectric domain structures. These features are consistent with a weakly ordered relaxor‐like state.

Temperature‐dependent X‐ray diffraction (XRD) patterns (Figure 8c) show that the material retains a pseudocubic perovskite structure from 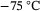 to 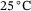 without observable peak splitting or symmetry changes. These results indicate suppression of a sharp structural phase transition and confirm structural stability over a broad temperature range.

The dielectric response (Figure 8d) exhibits a broad and frequency‐dependent permittivity peak characteristic of a diffuse phase transition. The extracted diffuseness parameter (γ≈1.8) indicates strong deviation from a normal ferroelectric transition and is consistent with relaxor‐like behavior. Such diffuse transition behavior enables gradual polarization evolution with temperature, thereby broadening the operational temperature span of the EC response [37].

The polarization behavior (Figure 8e) further supports this mechanism. Slim P–E hysteresis loops with small remanent polarization ($P_{r}$) and relatively large maximum polarization ($P_{m}$) are observed over the investigated temperature range. This behavior indicates strong field‐induced polarization with reduced hysteresis loss, favoring reversible EC operation and high energy efficiency [38].

According to Landau theory, under the assumptions that the lattice entropy is field independent and that the Landau parameter varies only slightly among perovskite‐structured ferroelectrics, the magnitude of the dipolar entropy change associated with the EC effect is correlated with $P_{m}^{2}-P_{r}^{2}$ [17], where $P_{m}$ and $P_{r}$ represent the maximum and remanent polarization, respectively. This relationship has been experimentally validated using measured entropy changes and polarization values for multiple ${\mathrm{BaTiO}}_{3}$‐based bulk ceramic compositions [17]. As shown in Figure 8f, the optimized material exhibits a favorable combination of large polarization response and low remanence compared with literature‐reported systems measured at similar temperatures. This polarization behavior contributes directly to the enhanced low‐temperature EC performance of the optimized composition. Abbreviations used for literature materials are summarized in Table S5.

To further rationalize why the optimized composition achieves a favorable balance between low‐temperature EC strength and operational temperature span, we compared its position with literature‐reported low‐temperature EC ceramics in the feature space, as discussed in the Supporting Information. The optimized composition is located in a favorable feature‐space region where the ferroelectric transition is sufficiently suppressed and broadened while sufficient field‐induced polarization is retained.

Overall, the coexistence of nanoscale structural disorder and suppressed long‐range ferroelectric ordering enables reversible field‐induced polarization over a broad temperature range. This mechanism explains the simultaneously enhanced EC strength and operational temperature span achieved in materials identified through the multi‐fidelity multi‐objective active‐learning framework.

## 3 Conclusion

In this work, a multi‐fidelity learning framework was developed to address heterogeneous EC datasets arising from direct and indirect measurement methods. By explicitly modeling the correlation and discrepancy between fidelity levels, the co‐kriging approach enables effective integration of sparse high‐fidelity data with abundant low‐fidelity data, resulting in substantially improved predictive accuracy and robustness compared with single‐fidelity and naively combined models. Building on this framework, a multi‐objective active learning strategy was employed to optimize low‐temperature EC strength and operational temperature span. Incorporation of low‐fidelity information improves both predictive performance and exploration efficiency, enabling faster convergence toward optimal regions of the composition–processing space, as demonstrated by cross‐validation and simulated search scenarios. Guided by the proposed framework, a multi‐element‐doped ${\mathrm{BaTiO}}_{3}$‐based ferroelectric ceramic, $\left({\mathrm{Ba}}_{0.93}{\mathrm{Ca}}_{0.06}{\mathrm{Sr}}_{0.01}\right)\left({\mathrm{Ti}}_{0.75}{\mathrm{Zr}}_{0.19}{\mathrm{Sn}}_{0.01}{\mathrm{Hf}}_{0.05}\right)O_{3}$, was identified, exhibiting an EC strength of $0.06\times 10^{-6}$ K·m/V at 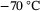 together with a broad operational temperature span of 75 K. Experimental characterization indicates that the enhanced performance originates from a suppressed and diffuse phase transition associated with a relaxor‐like or weakly ordered state, enabling broad temperature stability together with large reversible polarization. Overall, this work demonstrates that combining multi‐fidelity learning with data‐efficient optimization provides an effective strategy for accelerating materials discovery under realistic experimental constraints. The framework is particularly suited to functional materials systems with sparse and heterogeneous datasets and is readily extendable beyond electrocaloric ceramics.

## 4 Experimental Section

${\mathrm{BaTiO}}_{3}$‐based bulk ceramics were selected as the model material system because of the availability of relatively large EC datasets together with their practical advantages of lead‐free composition and scalable fabrication [26, 39, 40].

Designed ${\mathrm{BaTiO}}_{3}$‐based ferroelectric ceramics were synthesized using a conventional solid‐state reaction route. High‐purity starting powders, including ${\mathrm{BaCO}}_{3}$, ${\mathrm{CaCO}}_{3}$, ${\mathrm{SrCO}}_{3}$, ${\mathrm{TiO}}_{2}$, ${\mathrm{BaZrO}}_{3}$, ${\mathrm{SnO}}_{2}$, and ${\mathrm{HfO}}_{2}$, were weighed according to target compositions, ball‐milled in alcohol for 6 h, dried at 70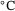, and calcined at 1250 or 1300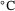 for 3 h. After re‐milling and binder addition, the powders were uniaxially pressed into pellets and sintered at 1400 or 1450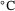 for 3 h. The sintered samples were polished and coated with silver electrodes for electrical measurements.

Temperature‐dependent dielectric permittivity was measured using an LCR meter (HIOKI 3532). Phase structures were characterized by temperature‐dependent X‐ray diffraction (XRD, Bruker D8 ADVANCE, Cu $K_{\alpha }$ radiation) equipped with an in situ cooling stage. Microstructures were characterized using a JEM‐F200 transmission electron microscope (TEM).

Electrocaloric performance was evaluated by direct measurements using a commercial EC measurement system (GoGo Instrument), where the adiabatic temperature change (ΔT) was measured under applied electric fields. Further details of sample preparation, EC measurements, and data processing are provided in the Supporting Information.

## Conflicts of Interest

The authors declare no conflicts of interest.

## Supporting information


**Supporting File**: advs77152‐sup‐0001‐SuppMat.pdf

## Data Availability

All the data and codes used to reproduce the results are available at https://github.com/matermlr/Fusing‐direct‐and‐indirect‐electrocaloric‐measurements. Including the measured EC data for the newly synthesized compositions (Section 2.4) and the composition–processings identified during the active‐learning optimization (Section 2.6).

## References

[1] F. Du , T. Yang , H. Hao , et al., “Giant Electrocaloric Effect in High‐Polar‐Entropy Perovskite Oxides,” Nature 640, no. 8060 (2025): 924–930, 10.1038/s41586-025-08768-8. 40205056

[2] B. Wu , H. Tao , K. Chen , et al., “Giant Intrinsic Electrocaloric Effect in Ferroelectrics by Local Structural Engineering,” Nature Communications 16, no. 1 (2025): 7515, 10.1038/s41467-025-61860-5. PMC12350644 40804249

[3] A. Torell'o , P. Lheritier , T. Usui , et al., “Giant Temperature Span in Electrocaloric Regenerator,” Science 370, no. 6512 (2020): 125–129, 10.1126/science.abb8045. 33004522

[4] World Health Organization , “Ultra‐Low Temperature Storage and Transport for Vaccines: An Overview of Options and Challenges,” (World Health Organization, February 2021): accessed April 25, 2026, https://www.who.int/docs/default‐source/coronaviruse/act‐accelerator/covax/who_covid‐19_ultracoldchain_overview_en‐%281%29.pdf.

[5] M. O. Jusu , G. Glauser , J. F. Seward , et al., “Rapid Establishment of a Cold Chain Capacity of ‐60 or Colder for the STRIVE Ebola Vaccine Trial During the Ebola Outbreak in Sierra Leone,” The Journal of Infectious Diseases 217, no. S1 (2018): S48–S55, 10.1093/infdis/jix336. 29788339 PMC6927859

[6] X. Wei , X. Lv , A. Cui , et al., “Novel 3‐3‐Like Heterostructure Engineering Enables ${\mathrm{BaTiO}}_{3}$‐Based Ferroelectric Ceramics with Superior Electrocaloric Performance,” Acta Materialia 285 (2025): 120674, 10.1016/j.actamat.2024.120674.

[7] B. Nair , T. Usui , S. Crossley , et al., “Large Electrocaloric Effects in Oxide Multilayer Capacitors Over a Wide Temperature Range,” Nature 575, no. 7783 (2019): 468–472, 10.1038/s41586-019-1634-0. 31597164

[8] B. Neese , B. Chu , S.‐G. Lu , Y. Wang , E. Furman , and Q. M. Zhang , “Large Electrocaloric Effect in Ferroelectric Polymers Near Room Temperature,” Science 321, no. 5890 (2008): 821–823, 10.1126/science.1159655. 18687960

[9] J. Mei , M. Bai , Y. Gao , et al., “Large Electrocaloric Temperature Change in Lead‐Free (${\mathrm{Bi}}_{0.47}{\mathrm{Na}}_{0.47}{\mathrm{Ba}}_{0.06}$)${\mathrm{TiO}}_{3}$‐Based Ceramics via Regulating Non‐Ergodic ‐ Ergodic Transition,” Chemical Engineering Journal 522 (2025): 166930, 10.1016/j.cej.2025.166930.

[10] Z. Liu , M. Bai , H. Tan , Y. Ling , and Z. Cao , “Experimental Test on the Performance of a ‐80 Cascade Refrigeration Unit Using Refrigerants R290‐R170 for COVID‐19 Vaccines Storage,” Journal of Building Engineering 63 (2023): 105537, 10.1016/j.jobe.2022.105537.

[11] Y. Meng , Z. Zhang , H. Wu , et al., “A Cascade Electrocaloric Cooling Device for Large Temperature Lift,” Nature Energy 5, no. 12 (2020): 996–1002, 10.1038/s41560-020-00715-3.

[12] J. Shi , D. Han , Z. Li , et al., “Electrocaloric Cooling Materials and Devices for Zero‐Global‐Warming‐Potential, High‐Efficiency Refrigeration,” Joule 3, no. 5 (2019): 1200–1225, 10.1016/j.joule.2019.03.021.

[13] X. Moya , E. Stern‐Taulats , S. Crossley , et al., “Giant Electrocaloric Strength in Single‐Crystal ${\mathrm{BaTiO}}_{3}$ ,” Advanced Materials 25, no. 9 (2013): 1360–1365, 10.1002/adma.201203823. 23307287

[14] Y. Nouchokgwe , P. Lheritier , C.‐H. Hong , et al., “Giant Electrocaloric Materials Energy Efficiency in Highly Ordered Lead Scandium Tantalate,” Nature Communications 12, no. 1 (2021): 3298, 10.1038/s41467-021-23354-y. PMC8172889 34078891

[15] W. Xiao , Y. Wu , Y. Liu , et al., “Enhanced Electrocaloric Effect in Ferroelectric Ceramics via Defect Dipole Engineering,” Nature Communications 16, no. 1 (2025): 8909, 10.1038/s41467-025-63963-5. PMC12504552 41057335

[16] X. Wei , K. Zeng , X. Shi , et al., “Multiscale‐Engineered Ferroelectric Ceramics Exhibiting Superior Electrocaloric Performance,” Nature Communications 16, no. 1 (2025): 8851, 10.1038/s41467-025-63909-x. PMC12494770 41044105

[17] R. Yuan , Z. Liu , Y. Xu , et al., “Optimizing Electrocaloric Effect in Barium Titanate‐Based Room Temperature Ferroelectrics: Combining Landau Theory, Machine Learning and Synthesis,” Acta Materialia 235 (2022): 118054, 10.1016/j.actamat.2022.118054.

[18] J. Gong , S. Chu , R. K. Mehta , and A. J. H. McGaughey , “XGBoost Model for Electrocaloric Temperature Change Prediction in Ceramics,” npj Computational Materials 8, no. 1 (2022): 140, 10.1038/s41524-022-00826-3.

[19] X. Xu , M. Li , X. Lin , and H. Wang , “Giant Reversible Electrocaloric Effect in Monolayer Group‐IV Monochalcogenides,” Physical Review B 110 (2024): 064103, 10.1103/PhysRevB.110.064103.

[20] R. Yuan , B. Wang , J. Li , et al., “Machine Learning‐Enabled Design of Ferroelectrics with Multiple Properties via a Landau Model,” Acta Materialia 286 (2025): 120760, 10.1016/j.actamat.2025.120760.

[21] M. Nayak , B. Haldar , H. Joardar , et al., “Machine Learning Assisted Electrocaloric and Pyroelectric Performance of ${\mathrm{Ba}}_{0.85}{\mathrm{Ca}}_{0.15}{\mathrm{Ti}}_{0.9}{\mathrm{Zr}}_{0.1}O_{3}$ Ceramic for Solid State Refrigeration,” Ceramics International 51, no. 27, Part A (2025): 51803–51817, 10.1016/j.ceramint.2025.08.392.

[22] M. C. Bayir and E. Mensur , “XGBoost‐Based Modeling of Electrocaloric Property: A Bayesian Optimization in BCZT Electroceramics,” Materials 18, no. 12 (2025): 2682, 10.3390/ma18122682. 40572814 PMC12194719

[23] M. Su , R. Grimes , S. Garg , D. Xue , and P. V. Balachandran , “Machine‐Learning‐Enabled Prediction of Adiabatic Temperature Change in Lead‐Free ${\mathrm{BaTiO}}_{3}$‐Based Electrocaloric Ceramics,” ACS Applied Materials & Interfaces 13, no. 45 (2021): 53475–53484, 10.1021/acsami.1c15021. 34704727

[24] X. Moya , S. Kar‐Narayan , and N. D. Mathur , “Caloric Materials Near Ferroic Phase Transitions,” Nature Materials 13, no. 5 (2014): 439–450, 10.1038/nmat3951. 24751772

[25] Y. Liu , J. F. Scott , and B. Dkhil , “Direct and Indirect Measurements on Electrocaloric Effect: Recent Developments and Perspectives,” Applied Physics Reviews 3, no. 3 (2016): 031102, 10.1063/1.4958327.

[26] M. Krupska‐Klimczak , M. Frontczak , Z. 'Swierczy'nski , S. Semenov , D. Kajewski , and I. Jankowska‐Sumara , “Electrocaloric Effect on Lead‐Free Ferroelectrics: Challenges in Identifying Trends and Evaluating Predictive Models,” Materials 18, no. 19 (2025): 4444, 10.3390/ma18194444. 41095271 PMC12526495

[27] J. Li , D. Zhang , S. Qin , et al., “Large Room‐Temperature Electrocaloric Effect in Lead‐Free ${\mathrm{BaHf}}_{x}{\mathrm{Ti}}_{1-x}O_{3}$ Ceramics Under Low Electric Field,” Acta Materialia 115 (2016): 58–67, 10.1016/j.actamat.2016.05.044.

[28] X. Li , S.‐G. Lu , X.‐Z. Chen , H. Gu , X.‐S. Qian , and Q. M. Zhang , “Pyroelectric and Electrocaloric Materials,” Journal of Materials Chemistry C 1 (2013): 23–37, 10.1039/C2TC00283C.

[29] S. G. Lu , B. Rožič , Q. M. Zhang , et al., “Comparison of Directly and Indirectly Measured Electrocaloric Effect in Relaxor Ferroelectric Polymers,” Applied Physics Letters 97, no. 20 (2010): 202901, 10.1063/1.3514255.

[30] B. Wang , Y. Xu , Y. Zhou , and D. Xue , “Multi‐Fidelity Learning in Materials Informatics: Methodologies, Applications, and Outlook,” Journal of Materials Informatics 6, no. 16 (2026), 10.20517/jmi.2025.85.

[31] A. I. Forrester , A. Sóbester , and A. J. Keane , “Multi‐fidelity optimization via surrogate modelling,” Proceedings of the Royal Society A: Mathematical, Physical and Engineering Sciences 463, no. 2088 (2007): 3251–3269, 10.1098/rspa.2007.1900.

[32] L. Le Gratiet , “Multi‐Fidelity Gaussian Process Regression for Computer Experiments,” (2023).

[33] X.‐S. Qian , H.‐J. Ye , Y.‐T. Zhang , et al., “Giant Electrocaloric Response Over A Broad Temperature Range in Modified ${\mathrm{BaTiO}}_{3}$ Ceramics,” Advanced Functional Materials 24, no. 9 (2014): 1300–1305, 10.1002/adfm.201302386.

[34] R. Yin , J. Li , X. Su , et al., “Emergent Enhanced Electrocaloric Effect Within Wide Temperature Span in Laminated Composite Ceramics,” Advanced Functional Materials 32, no. 5 (2022): 2108182, 10.1002/adfm.202108182.

[35] Z. Kutnjak and B. Rožič , Indirect and Direct Measurements of the Electrocaloric Effect (Springer, 2014), 147–182.

[36] B. Rožič , B. Malič , H. Uršič , et al., “Direct Measurements of the Giant Electrocaloric Effect in Soft and Solid Ferroelectric Materials,” Ferroelectrics 405, no. 1 (2010): 26–31, 10.1080/00150193.2010.482884.

[37] Y. Huang , L. Zhang , P. Ge , et al., “Unveiling a Giant Electrocaloric Effect at Low Electric Fields Through Continuous Phase Transition Design,” Advanced Powder Materials 3, no. 5 (2024): 100225, 10.1016/j.apmate.2024.100225.

[38] E. Defay , S. Crossley , S. Kar‐Narayan , X. Moya , and N. D. Mathur , “The Electrocaloric Efficiency of Ceramic and Polymer Films,” Advanced Materials 25, no. 24 (2013): 3337–3342, 10.1002/adma.201300606. 23666863

[39] Y. Tang , X. Niu , Y. Jiang , et al., “ ${\mathrm{BaTiO}}_{3}$‐Based Electrocaloric Materials–Recent Progresses and Perspective,” Materials 18, no. 17 (2025): 4190, 10.3390/ma18174190. 40942616 PMC12430785

[40] X. Zhao , Z. Zhou , B. Liang , and S. Lu , “Large Room‐Temperature Electrocaloric Effect in Lead‐Free Relaxor Ferroelectric Ceramics With Wide Operation Temperature Range,” Materials 17, no. 21 (2024): 5241, 10.3390/ma17215241. 39517517 PMC11547186

