# Supplementary material for: Fusing Direct and Indirect Measurements Through Multi‐Fidelity Learning For Accelerated Electrocaloric Materials Discovery
Source: Adv Sci (Weinh). 2026 Sep 3:e77152. Online ahead of print. doi: 10.1002/advs.77152 (PMC13542402; doi:10.1002/advs.77152)
Supplement: Supplementary file 1 [file ADVS-9999-e77152-s001.pdf]

Supporting Information

Fusing direct and indirect measurements through multi-fidelity learning for accelerated electrocaloric materials discovery

Bo Wang,[1] Pengfei Dang,[1] Yuan Tian,[2] Zhengwang He,[1] Wei Gu,[1] Lixue Zhang,[1] Turab Lookman,[1] Yumei Zhou,[1] and Dezhen Xue[1, a)]

1)State Key Laboratory for Mechanical Behavior of Materials, Xi'an Jiaotong University, Xi'an 710049, China

2) College of Materials Science and Engineering, Chongqing University, Chongqing, 400045, China.

a) Electronic mail: xuedezhen@xjtu.edu.cn

A. Brief introduction of co-kriging algorithm

In this work, the multi-fidelity data modeling was realized by co-kriging algorithm. Co-kriging is the extension of kriging (Gaussian process regression), which can flexibly interpolate data and simultaneously provide model uncertainty. In the context of kriging, the target properties of data are regarded as realizations of a Gaussian random field $y(x)$, which can be completely characterized by its mean function $\mu(x)$ and its covariance function $k(x, \tilde{x})$. With the Markov property assumption, co-kriging model was proposed as[1]:

$$y_{HF}(x) = \rho\, y_{LF}(x) + y_d(x), \tag{S1}$$

where $y_{HF}(x)$ is the high-fidelity random field and $y_{LF}(x)$ is the low-fidelity random field, ρ represents a scale factor, $y_d(x)$ is an independent random field, that is, low-fidelity model and data can be corrected to high-fidelity counterpoints by scaling and plus a deviation term. Assuming the training datasets: low-fidelity $(\mathbf{X}_{LF}, \mathbf{y}_{LF})$ and high-fidelity $(\mathbf{X}_{LF}, \mathbf{y}_{LF})$, $\mathbf{x}$ is m-dimensions, then the co-kriging predictions (mean μ and variance σ^2) at new point $\mathbf{x}^*$ with the high-fidelity level are[2, 3]:

$$\mu(\mathbf{x}^*) = \hat{\mu}_{HF}(\mathbf{x}^*) + \mathbf{k}^{\mathrm{T}}\mathbf{K}^{-1}\left(\begin{pmatrix}\mathbf{y}_{LF}\\ \mathbf{y}_{HF}\end{pmatrix} - \begin{pmatrix}\hat{\mu}_{LF}(\mathbf{X}_{LF})\\ \hat{\mu}_{HF}(\mathbf{X}_{HF})\end{pmatrix}\right), \tag{S2}$$

$$\sigma^2(\boldsymbol{x}^*) = \hat{\sigma}^2_{HF} - \boldsymbol{k}^T\boldsymbol{K}^{-1}\boldsymbol{k}, \tag{S3}$$

where, $\hat{\mu}_{HF}(\bullet) = \hat{\rho}\hat{\mu}_{LF}(\bullet) + \hat{\mu}_d(\bullet)$, $\hat{\sigma}^2_{HF} = \hat{\rho}^2\hat{\sigma}^2_{LF} + \hat{\sigma}^2_d$. To balance the simplicity and flexibility, we assign a constant $\hat{\mu}_{LF}$ and a linear $\hat{\mu}_d(\mathbf{x})$. $\mathbf{K}$ and $\mathbf{k}$ stand for covariance matrices:

$$\mathbf{K} = \begin{pmatrix} k_{LF}(\mathbf{X}_{LF}, \mathbf{X}_{LF}) & \hat{\rho}k_{LF}(\mathbf{X}_{LF}, \mathbf{X}_{HF}) \\ \hat{\rho}k_{LF}(\mathbf{X}_{HF}, \mathbf{X}_{LF}) & \hat{\rho}k_{LF}(\mathbf{X}_{HF}, \mathbf{X}_{HF}) + k_d(\mathbf{X}_{HF}, \mathbf{X}_{HF}) \end{pmatrix}, \tag{S4}$$

$$\mathbf{k} = \begin{pmatrix} \hat{\rho}k_{LF}(\mathbf{X}_{LF}, \mathbf{x}^*) \\ \hat{\rho}k_{LF}(\mathbf{X}_{HF}, \mathbf{x}^*) + k_d(\mathbf{X}_{HF}, \mathbf{x}^*) \end{pmatrix}. \tag{S5}$$

Here, we use the power exponential covariance function:

$$k(\boldsymbol{x}, \boldsymbol{x}^*) = \hat{\sigma}^2 \exp(-\textstyle\sum_{j=1}^{m} \hat{\theta}_j \, ||x_j^* - x_j||^{\hat{p}_j}). \tag{S6}$$

Parameters $\hat{\mu}$, $\hat{\sigma}$, $\hat{\rho}$, $\hat{\theta}$ and $\hat{p}$ for the low-fidelity-level and the deviation term are obtained via maximum likelihood estimation based on the training datasets.

The single-fidelity kriging model adopts the same formulation and parameter inference procedure described above, while treating all training data as high-fidelity responses and retaining only the high-fidelity-level terms.

B. Feature definition and selection

For EC strength prediction, we considered three categories of features that may influence the output: composition, processing, and measurement conditions.

For composition-related features, rather than using elemental contents directly, we adopted physically meaningful descriptors computed as element fraction weighted averages, which improves interpretability and reduces dimensionality. Table S1 shows the physical features which describe A-site and B-site elements (atom or ions) of the perovskite ($BaTiO_3$). Previous studies have shown that these properties closely relate to the ferroelectrics properties[4-6].

TABLE S1. Physical features of elements (atom or ions) used in this work.

Symbol	Description	Reference
R	Shannon's (1976) ionic radius	[7]
t	Tolerance factor calculated by Shannon's ionic radii	[4]
tau	Modified tolerance factor	[8]
mu	Octahedral factor	[9]
BO	Bond-valence parameters in oxides	[10]
AR	Atomic radius	[11]
CR	Covalent radius	[12]
AV	Atomic volume	[4]
D	Ionic displacement	[4]
DC	Distance from core electron (Schubert)	[12]
DV	Distance from valence electron (Schubert)	[12]
EN	Electronegativity--absolute scale (Pearson 1988)	[13]
EN.P	Electronegativity--Pauling scale (Pauling 1960)	[14]
EN.MB	Electronegativity--Matyonov-Batsanov	[15]
P	Polarizability	[16]
EA	Ionization energies	[4, 11]
Zeff	Outermost electron effective nuclear charge	[17, 18]
HA	Heat of atomization	[19]
HF	Heat of fusion	[19]
TE	Coefficient of linear thermal expansion	[11]
MN	Mendeleev number	[20]
AN	Atomic number	
RAM	Relative atomic mass	
GR	Group in element period table	
PE	Period in element period table	
VZ	Ratio of valence electron number to nuclear charge	
D1	Density	
EC	Electrical conductivity	
NCT	$\in$ (+1, -1, 0), that captures the trend (increase, decrease or no change) of the dependence of the cubic to tetragonal ferroelectric transition temperature on the doping element	[4]
NTO	$\in$ (+1, -1, 0), that captures the trend (increase, decrease or no change) of the dependence of the tetragonal to orthorhombic ferroelectric transition temperature on the doping element	[4]

We described physical features of $BaTiO_3$ based ceramics as weighted fractions of the elemental features. Specifically, in order to reflecting the structural characteristics of $BaTiO_3$, we distinguish the dopant elements at A-site and B-site. For example, for $(Ba_{1-a-b}Ca_aSr_b)(Ti_{1-c-d-e}Zr_cSn_dHf_e)O_3$ and a feature X, we defined:

$$\mathrm{XA} = \mathrm{a} \times \mathrm{X}^{\mathrm{Ca}} + \mathrm{b} \times \mathrm{X}^{\mathrm{Sr}}, \tag{S7}$$

$$\mathrm{XB} = \mathrm{c} \times \mathrm{X}^{\mathrm{Zr}} + \mathrm{d} \times \mathrm{X}^{\mathrm{Sn}} + \mathrm{e} \times \mathrm{X}^{\mathrm{Hf}}, \tag{S8}$$

$$\mathrm{x} = \mathrm{XA} \times \mathrm{XB}, \tag{S9}$$

$$\mathrm{X} = \frac{\mathrm{XA}}{\mathrm{XB}}. \tag{S10}$$

That is, we appended A or B to present the features calculated for A or B site. We used lowercase to present the product of A and B site features, and used capital to present the ratio of A and B site features[4]. What need to be additionally explained in Table S1 are the definitions of t, tau, mu, NCT and NTO:

$$\mathrm{t} = \frac{\mathrm{RA+RO}}{\sqrt{2}(\mathrm{RB+RO})}, \tag{S11}$$

$$\mathrm{tau} = \frac{\mathrm{RO}}{\mathrm{RB}} - \mathrm{n_A}\left(\mathrm{n_A} - \frac{\mathrm{RA/RB}}{\ln(\mathrm{RA/RB})}\right), \tag{S12}$$

$$\mathrm{mu} = \frac{\mathrm{RB}}{\mathrm{RO}}, \tag{S13}$$

$$\mathrm{NCT} = \mathrm{a} \times 0 + \mathrm{b} \times (-1) + \mathrm{c} \times (-1) + \mathrm{d} \times (-1) + \mathrm{e} \times (-1), \tag{S14}$$

$$\mathrm{NTO} = \mathrm{a} \times (-1) + \mathrm{b} \times (-1) + \mathrm{c} \times 1 + \mathrm{d} \times 1 + \mathrm{e} \times 1. \tag{S15}$$

t, tau, mu are used to reflect the influence of doped elements on the crystal structure of barium titanate. In NCT and NTO, elements that raise the corresponding phase transition temperature are assigned a characteristic value of 1, elements that lower the corresponding phase transition temperature are assigned a characteristic value of -1, and elements for which the phase transition temperature shows no significant change with increasing element content are assigned a characteristic value of 0.

Composition-averaged descriptors simplify the local chemical environment and may not fully capture specific dopant interactions or short-range structural effects. Nevertheless, such descriptors have been widely used in machine-learning studies of

ferroelectric and dielectric ceramics and have demonstrated effectiveness in property prediction and composition design [6, 21-23]. In this work, additional descriptors describing relationships between different crystallographic sites, such as tolerance-factor-related features, were included to partially capture coupling effects. More sophisticated interaction-aware descriptors may further improve prediction capability in future studies. However, the focus of this work is to develop a multi-fidelity learning framework for integrating direct and indirect EC measurements rather than to establish a new descriptor family.

For processing-related features, we restricted our dataset and new synthesis to the conventional solid-state reaction route. Accordingly, we focused on the calcination temperature and dwell time, as well as the sintering temperature and dwell time, which are known to affect ceramic microstructure and, consequently, electrocaloric performance[24-28]. The processing features were defined as follows: "calcinT" for calcination temperature (°C), "calcint" for calcination dwell time (h), "cTdt" for calcination temperature divided by dwell time, "cTmt" for the product of scaled calcination temperature and scaled dwell time, "sinterT" for sintering temperature (°C), "sintert" for sintering dwell time (h), "sTdt" for sintering temperature divided by dwell time, and "sTmt" for the product of scaled sintering temperature and scaled dwell time.

For measurement conditions, the environmental temperature T and the electric-field change ΔE were included, as both are known to influence EC strength.

High-dimensional feature sets may introduce redundancy and increase model complexity, which can lead to overfitting and reduced generalization. Feature selection is therefore essential to improve model robustness, efficiency, and interpretability by retaining the key descriptors governing the target property. In this work, feature selection for EC strength was performed independently of the surrogate models (Kriging and co-Kriging). Using the high-fidelity direct-measurement dataset, we first evaluated feature importance with gradient boosting regression using all candidate features as inputs, which provides a relative importance score for each feature and ranks them accordingly (Fig. S1). We then selected features with relative importance greater than 1 and examined their pairwise correlations using Pearson correlation coefficients

to ensure that the retained features were not strongly correlated (Fig. S1). Based on this procedure, the final feature set used for EC strength surrogate modeling comprised ΔE, NCT, sTdt, sTmt, T, tau and cTdt.

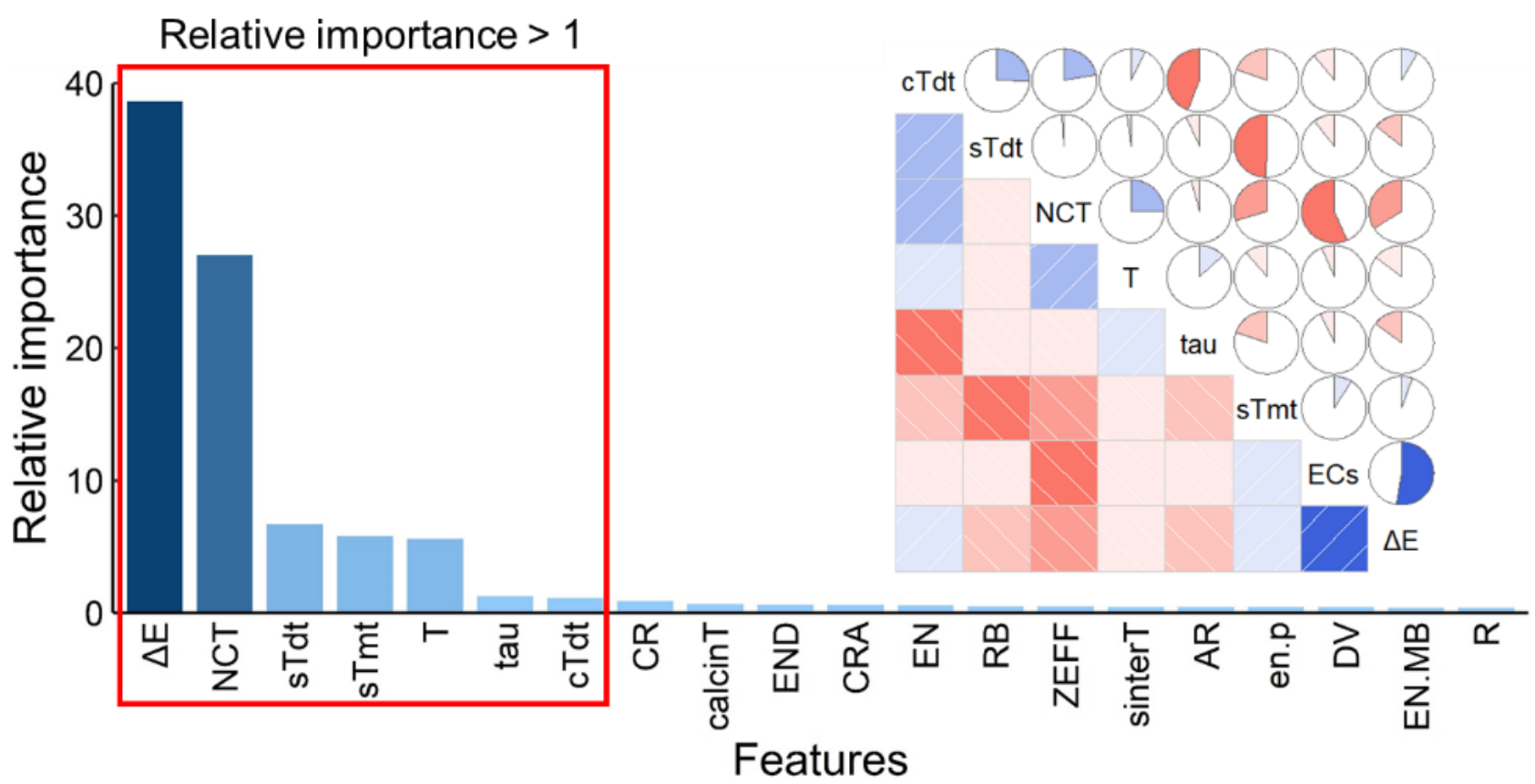

FIG. S1. Feature selection. Feature importance for EC strength ranked by gradient boosting regression (top 20 features) and Pearson correlation analysis of the selected features. Pairwise correlation coefficients are shown as a color-gradient heat map, and the filled fraction of each pie chart corresponds to the value of coefficient.

C. Expected improvement acquisition function

In Eq. (4) of the main text, the acquisition function components $F_{\Delta T/\Delta E}(\mathbf{x})$ and $F_{T_{span}}(\mathbf{x})$, corresponding to EC strength and temperature span, respectively, are defined using the expected improvement (EI), which balance exploration and exploitation by simultaneously considering the predicted value and its associated uncertainty[29]:

$$EI(\mu,\sigma) = \sigma[\phi(z) + z\Phi(z)] \tag{S16}$$

$$z = \frac{\mu-\mu^{**}}{\sigma} \tag{S17}$$

where μ and σ denote the predicted value and the associated uncertainty (standard deviation) of a material feature vector $\mathbf{x}$, respectively; μ^{**} denotes the maximum predicted value in the design space; and $\phi(z)$ and $\Phi(z)$ represent the probability density function and cumulative distribution function of the standard normal distribution, respectively.

The weight factors in Eq. (4) of the main text were set as follows: $w_1 = \frac{\sum F_{T_{span}}(x)}{\sum F_{\Delta T/\Delta E}(x)}$ and $w_2 = 1$, such that the acquisition-function components $F_{\Delta T/\Delta E}(\mathbf{x})$ and $F_{T_{span}}(\mathbf{x})$ were scaled to comparable ranges and contributed equally to the multi-objective acquisition function. The equal-contribution setting was selected because the design objective of this work was to simultaneously enhance low-temperature EC strength and broaden the operational temperature span. The experimentally validated improvement in both EC strength and operational temperature span demonstrates the practical utility of this balanced acquisition strategy. In principle, the weighting factors can be further adjusted to bias the search toward either higher peak EC response or broader temperature stability, providing flexibility for different application requirements.

D. Direct EC measurement

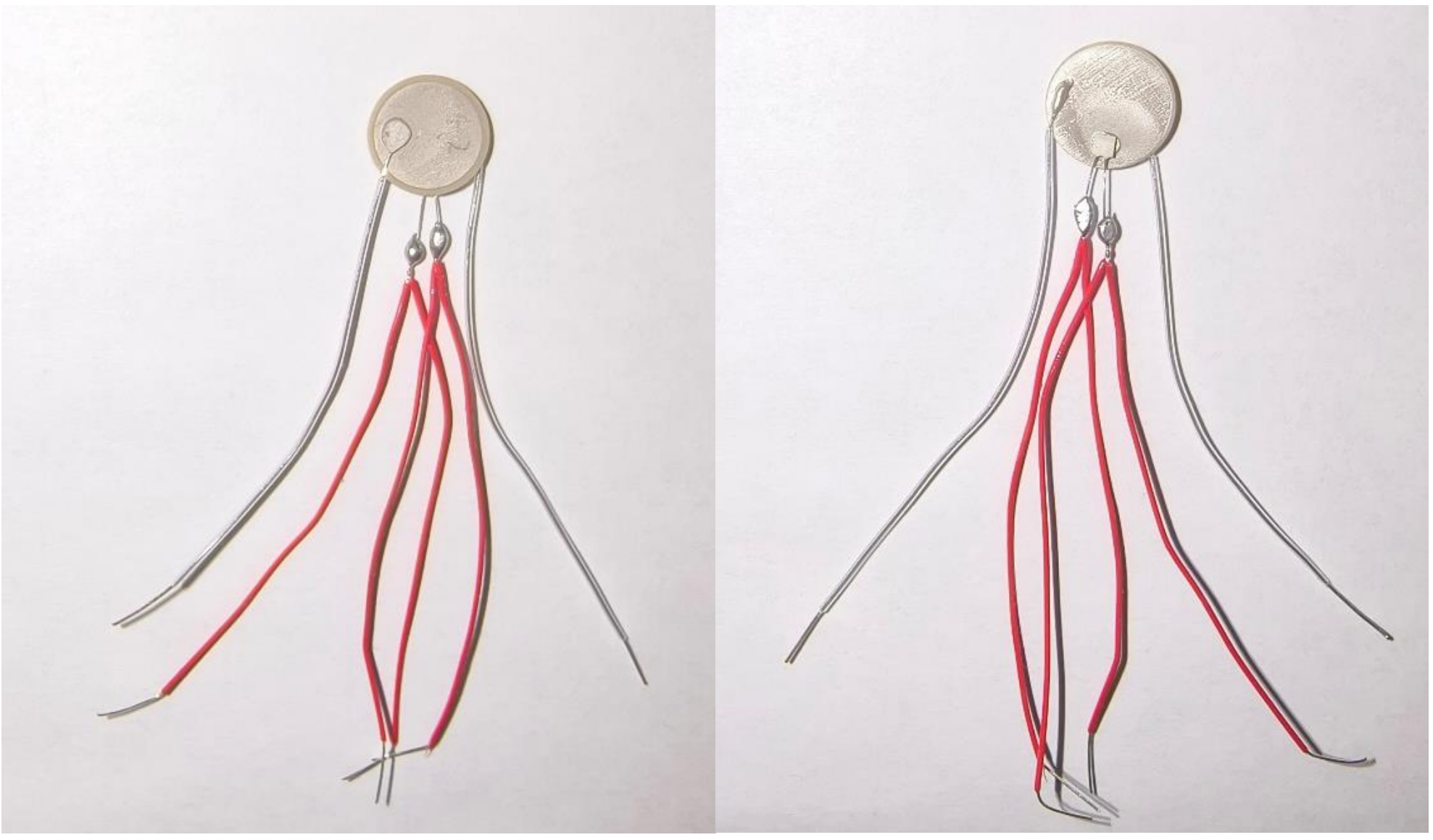

Fig. S2. Sample preparation for direct EC measurement. Silver electrodes were applied to both faces of the ceramic pellet. A 9-mm-diameter circular electrode was painted on one face (left panel), while the opposite face was fully covered with silver (right panel). The electrode overlap defines the active area, and the corresponding covered volume is the active part of the sample where the electrocaloric (EC) effect takes place. Teflon-insulated silver-coated copper wires were connected to the electrodes with a conductive silver adhesive for electric-field application. A PT100 sensor

was bonded to the electrode surface using conductive silver adhesive; its resistance was measured in a four-wire configuration and converted to temperature.

Owing to heat conduction and thermal equilibration among the ceramic and attached components, the measured temperature typically reaches its minimum several seconds after the electric field is switched off (Figure 7 (a) in the main text). Therefore, the temperature relaxation was fitted and extrapolated to $t = 0$ to obtain the instantaneous temperature change ΔT, using the following expression [30, 31]:

$$T(t) = T_{bath} + \Delta T e^{-t/\tau} \qquad \text{(S18)}$$

where $T(t)$ is the measured temperature at time t, t denotes the time elapsed after the electric field was removed, T_{bath} is the stabilized ambient temperature before the electric field was removed, and τ is the characteristic relaxation time obtained from the fitting.

Because heat is conducted and thermally equilibrated among the ceramic pellet and the attached components (electrodes, wires, and the temperature sensor), the measured temperature change ΔT represents an averaged response of the active and inactive parts. Therefore, the actual EC temperature change originating from the electroded (active) volume should be obtained by correcting for the heat capacity of the entire internally equilibrated measurement assembly [30, 32]:

$$\Delta T_{EC} = \Delta T \frac{C^p_{ceramic} + C^p_{wire} + C^p_{sensor}}{C^p_{EC}}$$

$$= \Delta T \frac{c^p_{ceramic} m_{ceramic} + c^p_{wire} m_{wire} + c^p_{sensor} m_{sensor}}{c^p_{EC} m_{EC}} \qquad \text{(S19)}$$

Here, C^p denotes the heat capacity of the corresponding part: the active part of the ceramic pellet ("EC"), the whole ceramic pellet ("ceramic"), the attached wires ("wire"), and the temperature sensor ("sensor"). The heat capacity of the silver electrodes was neglected because the electrode layer is thin and lightweight, and its contribution is therefore negligible. As an example, the room-temperature specific heat capacities of one ceramic pellet, the silver-coated copper wire, and the ceramic-encapsulated PT100

sensor are 0.42, 0.40, and 0.20 J/(g•K), respectively (measured by a Netzsch 214 differential scanning calorimeter). The masses of the ceramic pellet, wires, and sensor are 0.60, 0.050, and 0.016 g, respectively. The radii of the pellet and the smaller electrode are 5.5 and 4.6 mm, respectively. The correction factor is then calculated as $\frac{0.42\times0.6+0.4\times0.05+0.2\times0.016}{0.42\times0.6\times\frac{\pi\times4.6^2}{\pi\times5.5^2}} = 1.56$. The correction factors for all samples measured in this work range from 1.1 to 1.56, depending on the specific sample configuration.

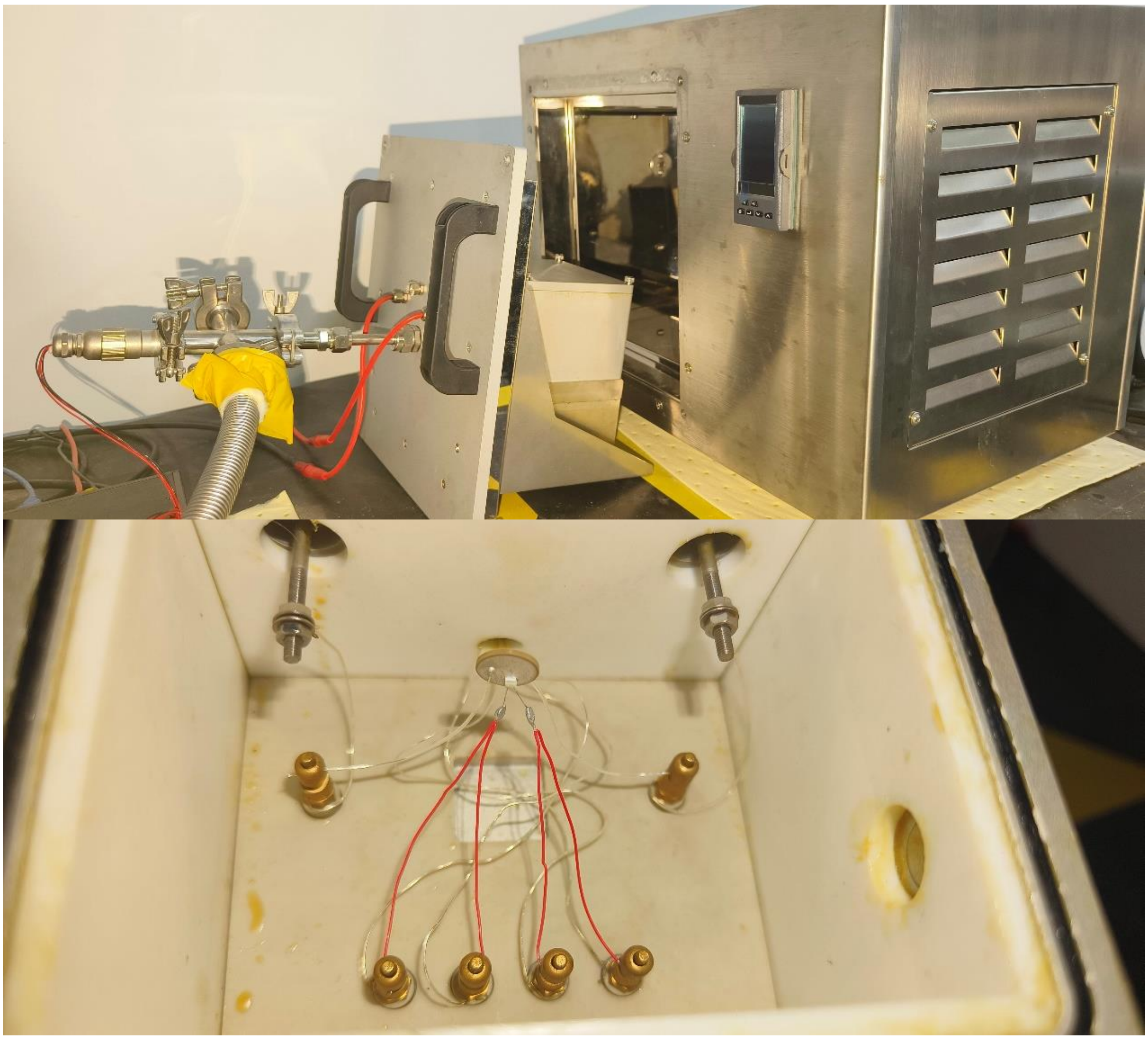

Fig. S3. Part of the direct EC measurement system.

A commercial direct electrocaloric (EC) measurement system (GoGo Instrument) was used to measure the quasi-adiabatic temperature change of the samples. The system comprised a high-voltage generator, a temperature chamber, a vacuum chamber, a turbomolecular pumping station (Pfeiffer HiCube 80 Eco), a digital multimeter

(Keithley 2000), a computer, and the corresponding control software. Fig. S3 (upper panel) shows a photograph of part of the direct EC measurement setup, including the temperature chamber, the vacuum chamber, and the connecting tube and wires to the other components. Fig. S3 (lower panel) shows the interior of the vacuum chamber. The silver wires attached to the sample and the temperature sensor were connected to binding posts, which also suspended the sample in air without contact with the chamber walls. During measurements, the vacuum chamber was placed inside the temperature chamber to control the ambient temperature. A high-vacuum condition of approximately 1×10^{-3} Pa was maintained in the vacuum chamber to achieve quasi-adiabatic conditions.

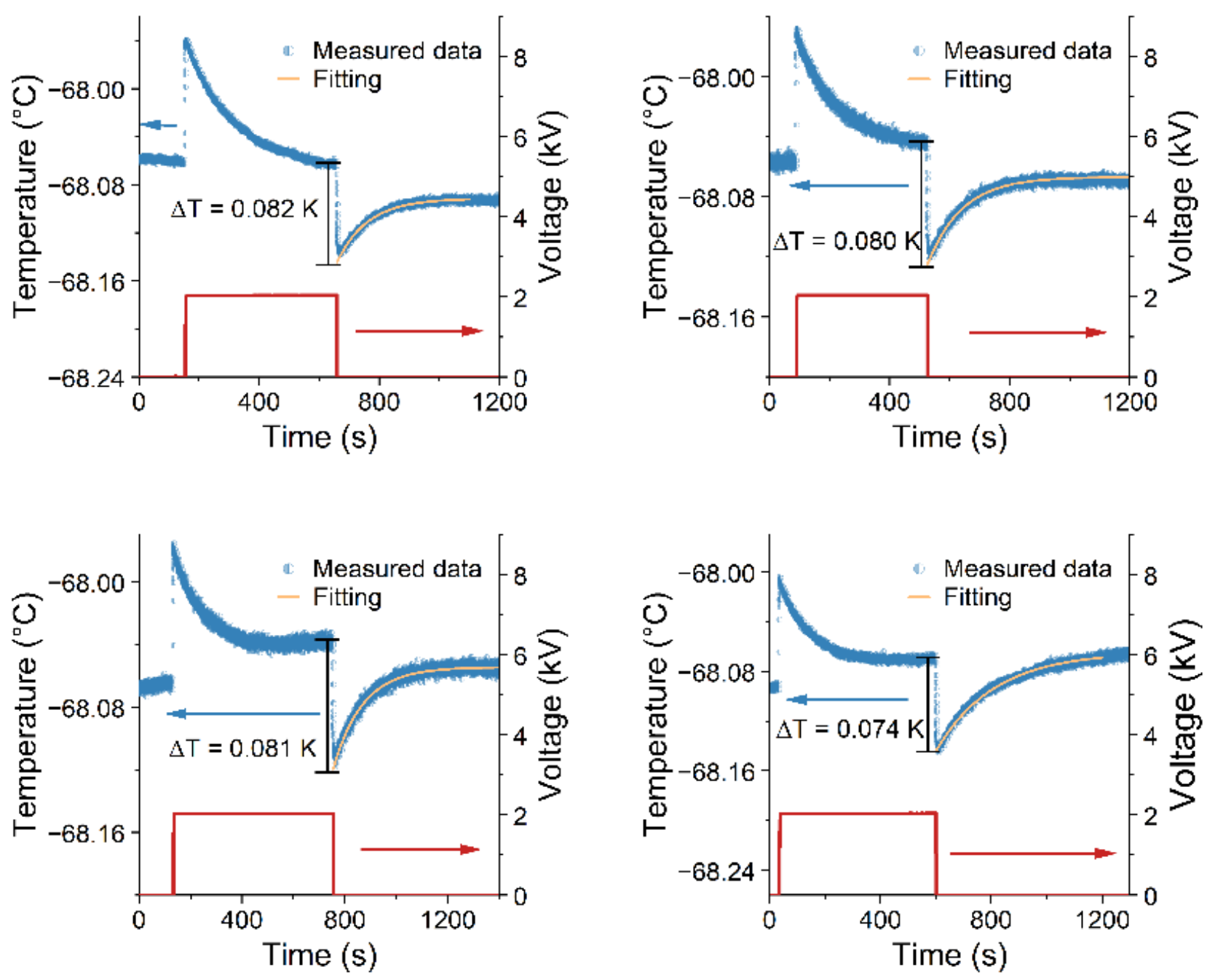

FIG. S4. Repeatability of direct electrocaloric measurements for the final optimized composition near −70 °C. Four repeated measurements are shown here, while the remaining measurement is presented in Fig. 7 of the main text. Blue symbols represent the measured temperature, orange lines represent the fitting temperature relaxation curves, and red curves show the applied voltage profile. All measurements were conducted at an electric-field change of 20 kV/cm.

To quantify the experimental uncertainty of the EC strength for the final optimized composition, five repeated direct EC measurements were conducted near -70 °C. Owing to the slight difference between the temperature-chamber reading and the local temperature measured by the sensor, the actual test temperature was -68.5 ± 0.5 °C. The measured EC strength values at 20 kV/cm were 0.062, 0.064, 0.058, 0.063, and 0.062 $\times10^{-6}$ K•m/V. The mean value and standard deviation were therefore 0.062 ± 0.002$\times10^{-6}$ K•m/V, indicating good repeatability of the direct EC measurement. The directly measured EC temperature changes from four repeated experiments are shown in Fig. S4, while the remaining measurement is presented in Fig. 7 of the main text.

E. Co-located data alignment

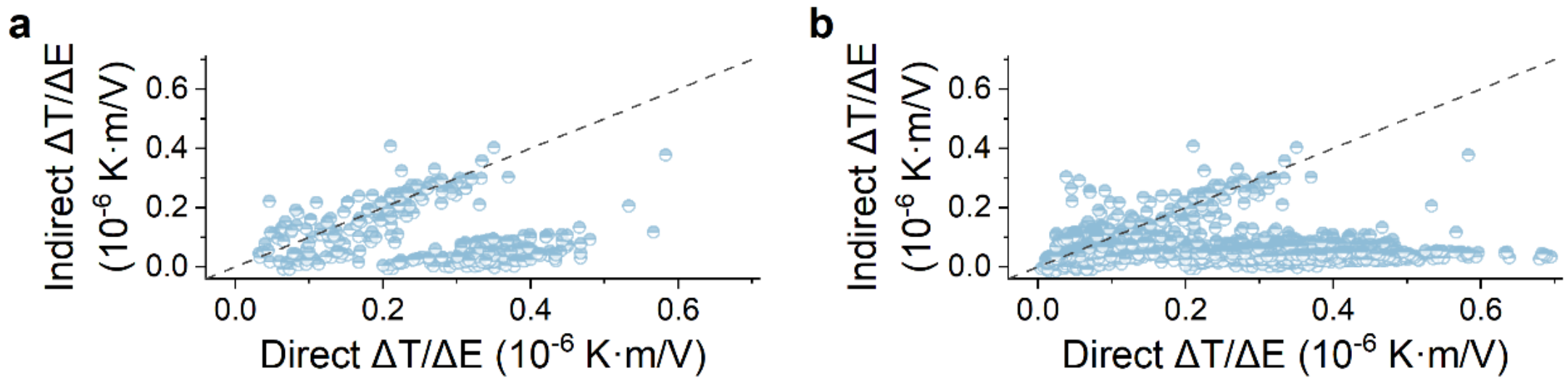

FIG. S5. Co-located data alignment. Co-located data, defined as data points with identical composition, processing parameters, and measurement conditions across fidelity levels, are required for learning the co-kriging correlation term [Eq. (3) in the main text]. Consequently, the number of co-located pairs constrains the number of valid direct (high-fidelity) data points available for model construction. To fully exploit the direct dataset, a data-alignment strategy was applied. Specifically, a Gaussian process regression model trained using indirect measurements was employed to estimate corresponding values at non-co-located high-fidelity positions, enabling partial augmentation of co-located data. (a) EC strength of the original co-located dataset, comprising 328 entries (identical to Fig. 3(a) in the main text). (b) EC strength of the augmented co-located dataset, comprising 1421 entries, corresponding to the full direct dataset. The close similarity in distribution between panels (a) and (b) demonstrates that the alignment strategy faithfully preserves the original data characteristics and serves only to meet the co-kriging requirement, without introducing a strong bias into model training.

The input features and output target used in this data-aligned model were kept identical to those used in the multi-fidelity and single-fidelity models, including composition descriptors, processing parameters, and measurement conditions as inputs, and EC strength as the output. Gaussian process regression was adopted because it is consistent with the co-kriging framework used throughout this work and can provide smooth interpolation for materials data. To reduce randomness in model training, which arises from the maximum likelihood estimation of model parameters, four independently trained Gaussian process models were constructed, and the mean of their predictions was used as the aligned low-fidelity value. Because the auxiliary model was used only to estimate aligned low-fidelity values at existing high-fidelity locations and not for independent performance evaluation, no separate validation procedure was conducted for this alignment step.

The auxiliary data-alignment procedure may introduce uncertainty because missing low-fidelity counterparts are estimated using an auxiliary model. To evaluate the potential influence of this approximation, the feature-space distributions of the original strictly co-located dataset and the aligned dataset were compared (Fig. S5). Their close similarity indicates that the alignment procedure does not introduce an obvious distributional shift. The aligned values were used only as low-fidelity counterparts required for co-kriging construction and were not used as validation or test labels. Model evaluation was performed exclusively against experimentally measured high-fidelity data. Nevertheless, the aligned values remain dependent on the auxiliary model, and future work may further quantify this uncertainty through uncertainty propagation within the multi-fidelity framework.

F. Newly synthesized materials for validating the models

The predictive capability of the models was assessed using newly synthesized samples. To ensure physically meaningful compositions, the candidate space was constrained to non-toxic dopant elements and composition ranges satisfying the tolerance-factor criterion for suppressing secondary phase formation. The resulting design space is described by $(Ba_{1-x-y}Ca_xSr_y)(Ti_{1-u-v-w}Zr_uSn_vHf_w)O_3$, with $0.65 \leq 1 - x$

– y, x ≤ 0.25, y ≤ 0.1, 0.75 ≤ 1 − u − v – w, u ≤ 0.25, v ≤ 0.15, and w ≤ 0.12. Twelve compositions were randomly selected from this space (Table S2). The directly measured EC responses of these samples are available in a public Github repository: https://github.com/diegoxue/Fusing-direct-and-indirect-electrocaloric-measurements.

TABLE S2. Newly synthesized materials for validating the models.

Composition
$Ba_{0.84}Ca_{0.06}Sr_{0.1}Ti_{0.89}Zr_{0.04}Sn_{0.07}O_3$
$Ba_{0.85}Ca_{0.15}Ti_{0.89}Zr_{0.08}Hf_{0.03}O_3$
$Ba_{0.88}Ca_{0.03}Sr_{0.09}Ti_{0.97}Sn_{0.03}O_3$
$Ba_{0.84}Ca_{0.06}Sr_{0.1}Ti_{0.89}Zr_{0.04}Sn_{0.07}O_3$
$Ba_{0.86}Ca_{0.11}Sr_{0.03}Ti_{0.87}Zr_{0.09}Sn_{0.01}Hf_{0.03}O_3$
$Ba_{0.8754}Ca_{0.1246}Ti_{0.89}Zr_{0.06}Sn_{0.05}O_3$
$Ba_{0.8988}Ca_{0.1012}Ti_{0.92}Zr_{0.01}Sn_{0.07}O_3$
$Ba_{0.84}Ca_{0.06}Sr_{0.1}Ti_{0.89}Zr_{0.04}Sn_{0.07}O_3$
$Ba_{0.85}Ca_{0.09}Sr_{0.06}Ti_{0.82}Zr_{0.05}Sn_{0.01}Hf_{0.12}O_3$
$Ba_{0.86}Ca_{0.14}Ti_{0.89}Zr_{0.03}Sn_{0.04}Hf_{0.04}O_3$
$Ba_{0.91}Ca_{0.07}Sr_{0.02}Ti_{0.86}Zr_{0.01}Sn_{0.02}Hf_{0.11}O_3$
$Ba_{0.65}Ca_{0.25}Sr_{0.1}Ti_{0.97}Zr_{0.01}Sn_{0.02}O_3$

G. Construction and optimization of the candidate space for low-temperature electrocaloric materials

The candidate space for optimizing low-temperature electrocaloric materials was defined as follows. The composition space followed the settings described above (Section F), with a composition increment of 0.01. In addition, only compositions with configurational entropy larger than R were retained, corresponding to medium- and high-entropy ceramics. This screening resulted in 583,272 candidate compositions.

Because the calcination temperature and sintering temperature can strongly influence the properties of ferroelectric ceramics, these two processing parameters were also included as optimization variables. Based on prior experimental experience, two levels were assigned for the calcination temperature, namely 1250 °C and 1300 °C, and two levels were assigned for the sintering temperature, namely 1400 °C and 1450 °C. Furthermore, to ensure consistency with the feature definition of the surrogate model,

testing conditions were also included in the candidate space. A moderate $\Delta E = 20\ \mathrm{kV/cm}$ was assigned. The test temperature range of the materials was set from -140 °C to 60 °C with an interval of 5 °C. In this way, the resulting virtual design space contained a total of 95,656,608 entries.

TABLE S3. Selected compositions and processing parameters from the active learning iterations.

Iter	Model	Composition	Sintering temperature (°C)	Calcination temperature (°C)
1	Multi-fidelity	$Ba_{0.9}Sr_{0.1}Ti_{0.75}Zr_{0.19}Sn_{0.04}Hf_{0.02}O_3$	1450	1250
	Direct-only	$Ba_{0.95}Ca_{0.01}Sr_{0.04}Ti_{0.76}Zr_{0.13}Sn_{0.06}Hf_{0.05}O_3$	1450	1300
2	Multi-fidelity	$Ba_{0.77}Ca_{0.13}Sr_{0.1}Ti_{0.75}Zr_{0.14}Sn_{0.1}Hf_{0.01}O_3$	1450	1300
	Direct-only	$Ba_{0.91}Ca_{0.08}Sr_{0.01}Ti_{0.81}Zr_{0.11}Sn_{0.03}Hf_{0.05}O_3$	1450	1300
3	Multi-fidelity	$Ba_{0.93}Ca_{0.06}Sr_{0.01}Ti_{0.75}Zr_{0.19}Sn_{0.01}Hf_{0.05}O_3$	1450	1250
	Direct-only	$Ba_{0.91}Ca_{0.01}Sr_{0.08}Ti_{0.75}Zr_{0.21}Hf_{0.04}O_3$	1400	1250

The trained multi-fidelity and direct-only surrogate models were then used to predict EC strength and the corresponding operational temperature span (see Section I) throughout the candidate space. The multi-objective active learning process followed the strategy described in Section 2.2 of the main text, and the selected compositions and processing parameters at each iteration are summarized in Table S3. Corresponding EC response measurements of are available in a public Github repository: https://github.com/diegoxue/Fusing-direct-and-indirect-electrocaloric-measurements.

H. Details for literature materials comparison

To provide a more complete assessment of low-temperature EC performance, we further collected representative literature data beyond lead-free bulk ceramics. Because this work targets biomedical storage and transport near -70 °C, materials were included when EC data were reported close to this temperature, approximately within -70 ± 10 °C, and when the operational temperature span could be obtained or estimated from the reported data. The expanded comparison is shown in Fig. S6. This broader comparison

shows that thin films and lead-containing systems can exhibit higher EC strength or broader temperature span than lead-free bulk ceramics. Nevertheless, the main comparison in the manuscript focuses on lead-free bulk ceramics because they provide the most relevant benchmark for the targeted application scenario and experimental configuration investigated in this work.

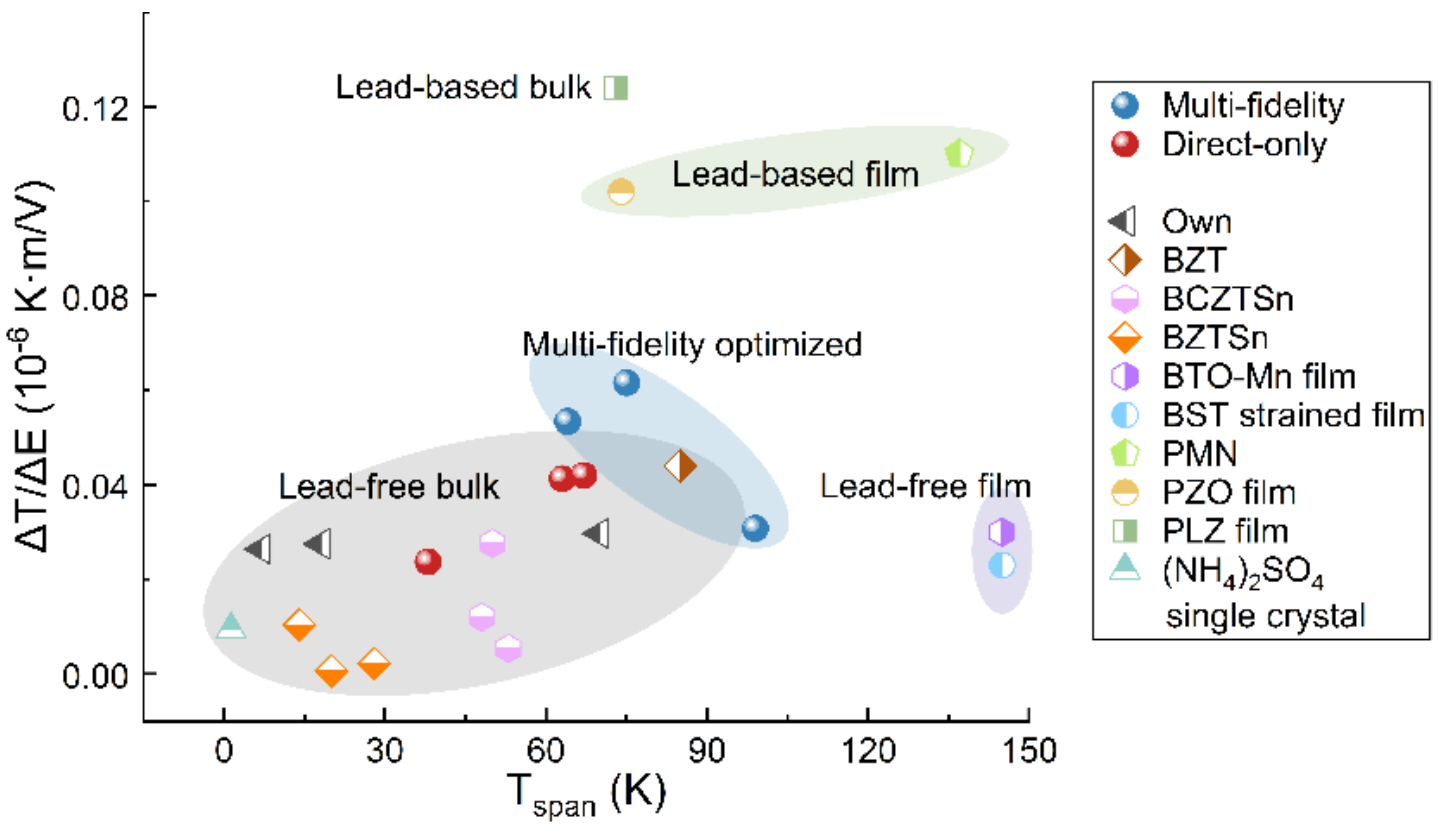

Fig. S6. Broader comparison of low-temperature electrocaloric materials measured near -70 °C. The comparison includes lead-free bulk ceramics, lead-containing systems, thin films, and single crystals when the EC response near the target temperature and the operational temperature span were available from the literature.

TABLE S4. Abbreviations used for literature-reported low-temperature electrocaloric materials corresponding to Fig. S6, Figure 7(e) and (f) of the main text. “Own” denotes the newly synthesized compositions for validation in this work (Section F).

Abbreviation	Composition	ΔE (kV/cm)	Reference
BZT	$BaTi_{0.65}Zr_{0.35}O_3$	20	[33]
BCZTSn	$Ba_{0.95}Ca_{0.05}Ti_{0.8325}Zr_{0.0925}Sn_{0.075}O_3$<br>$Ba_{0.9}Ca_{0.1}Ti_{0.8325}Zr_{0.0925}Sn_{0.075}O_3$<br>$Ba_{0.85}Ca_{0.15}Ti_{0.8325}Zr_{0.0925}Sn_{0.075}O_3$	8.7	[34]
BZTSn	$BaTi_{0.81}Zr_{0.09}Sn_{0.1}O_3$<br>$BaTi_{0.765}Zr_{0.085}Sn_{0.15}O_3$<br>$BaTi_{0.72}Zr_{0.08}Sn_{0.2}O_3$	8	[35]
BTO-Mn film	--	757.2	[36]
BST strained film	$Ba_{0.3}Sr_{0.7}TiO_3$	509	[37]
PMN	$Pb(Mg_{1/3}Nb_{2/3})O_3$	22	[38]
PZO film	$PbZrO_3$	600	[39]

PLZ film	$Pb_{0.97}La_{0.02}ZrO_3$	500	[40]
$(NH_4)_2SO_4$ single crystal	$(NH_4)_2SO_4$	11	[41]

TABLE S5. Abbreviations used for literature-reported bulk ceramics with low-temperature polarization data corresponding to Figure 8(f) of the main text.

Abbreviation	Composition	Reference
BTSn28	$BaTi_{0.72}Sn_{0.28}O_3$	[42]
BT-BNMT	$0.86BaTiO_3–0.14(Bi_{3/4}Na_{1/4})(Mg_{1/4}Ti_{3/4})O_3$	[43]
BZT	$BaTi_{0.7}Zr_{0.3}O_3$	[44]
BTSn11	$BaTi_{0.89}Sn_{0.11}O_3$	[45]
PZT	$Pb_{0.990}(ZrTi_{0.47}(Sb_{0.67}Nb_{0.33})_{0.08})O_3$	[46]
BT	$BaTiO_3$	[45]

I. Determination of the predicted temperature span

The surrogate model has a single output—the target property, electrocaloric strength (EC strength)—from which we derive the predicted temperature width and its associated uncertainty. Figure S6 shows an example of the predicted EC strength for a representative material. We consider a prediction temperature range from −140 to 60 °C (133–333 K) with a step of 5 K, which provides a reasonable balance between temperature resolution and computational cost. The data points and error bars denote the predicted EC strength and its uncertainty (assumed here as 10% standard deviation). The horizontal dashed line indicates 80% of the peak predicted EC strength; the temperature interval over which the predicted EC strength exceeds this threshold is defined as the predicted temperature width (a commonly used definition). Accordingly, the predicted width is obtained by counting the temperature points whose predicted values lie above the dashed line. The lower bound of the predicted width is determined by counting the temperature points whose lower error-bar bounds remain above the dashed line, while the upper bound is determined by counting the temperature points whose upper error-bar bounds are above the dashed line. The uncertainty of the predicted width is defined as: (Upper bound – Lower bound) / 2.

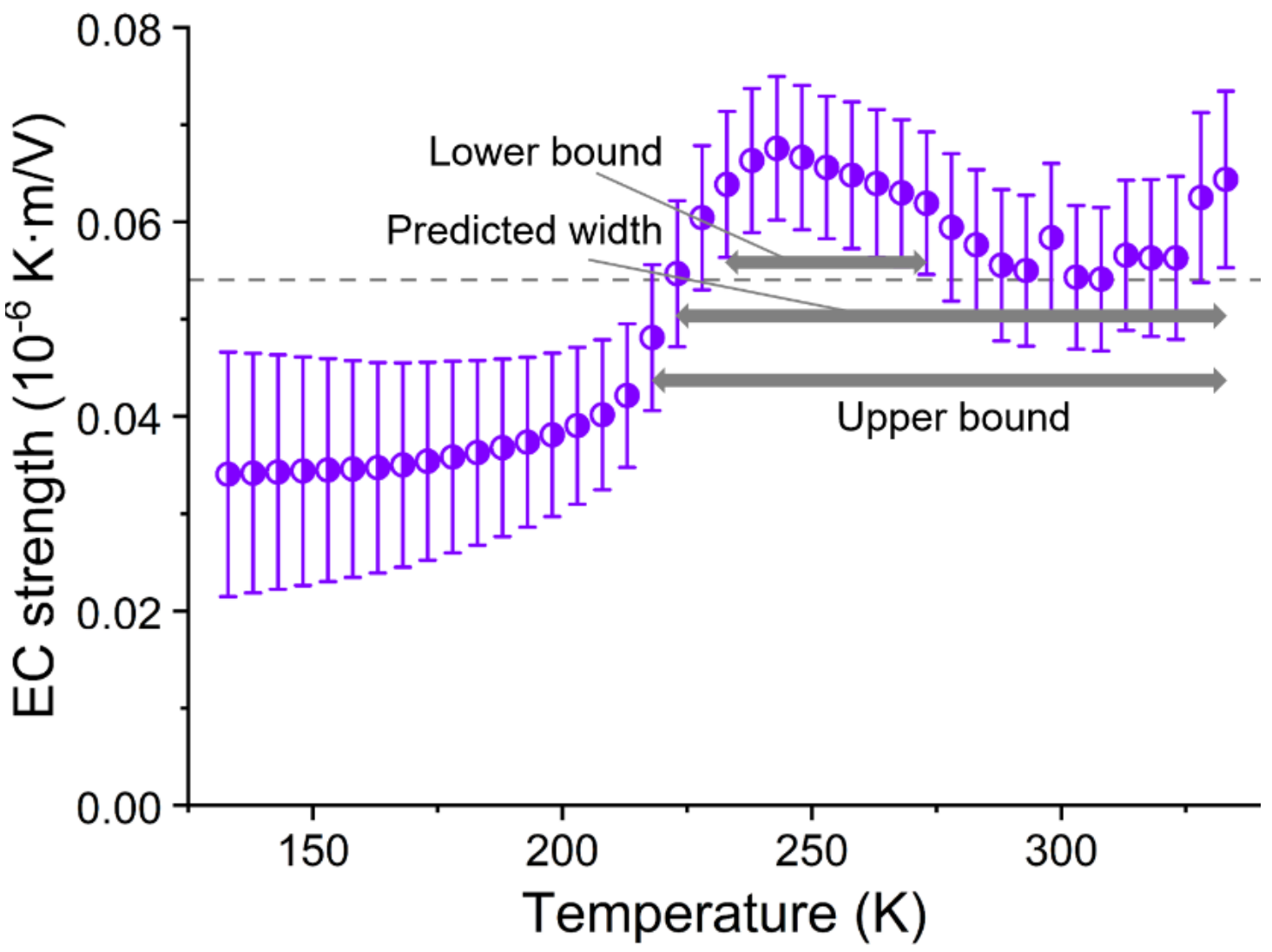

Fig. S7. Schematic illustration of how the predicted temperature width and its uncertainty are determined.

J. Feature-space interpretation of the optimized composition

To interpret the composition identified by the multi-fidelity active-learning framework, its position in the NCT-EN.P feature space was analyzed (Fig. S8). The identified composition resides in a balanced descriptor region associated with simultaneously enhanced EC strength and broad operational temperature span. The NCT descriptor reflects the influence of dopant substitution on the ferroelectric transition temperature of $BaTiO_3$. A moderately negative NCT value suggests a shift of the transition toward the low-temperature regime while avoiding excessive suppression that may reduce the polarization change required for strong EC response. The EN.P descriptor represents the electronegativity-related chemical environment introduced by dopant elements, and the relatively high EN.P value is consistent with enhanced chemical heterogeneity and the experimentally observed diffuse phase-transition behavior. Therefore, the identified composition achieves a balance between transition-temperature regulation and reversible polarization response, consistent with the observed relaxor-like behavior and enhanced low-temperature EC performance.

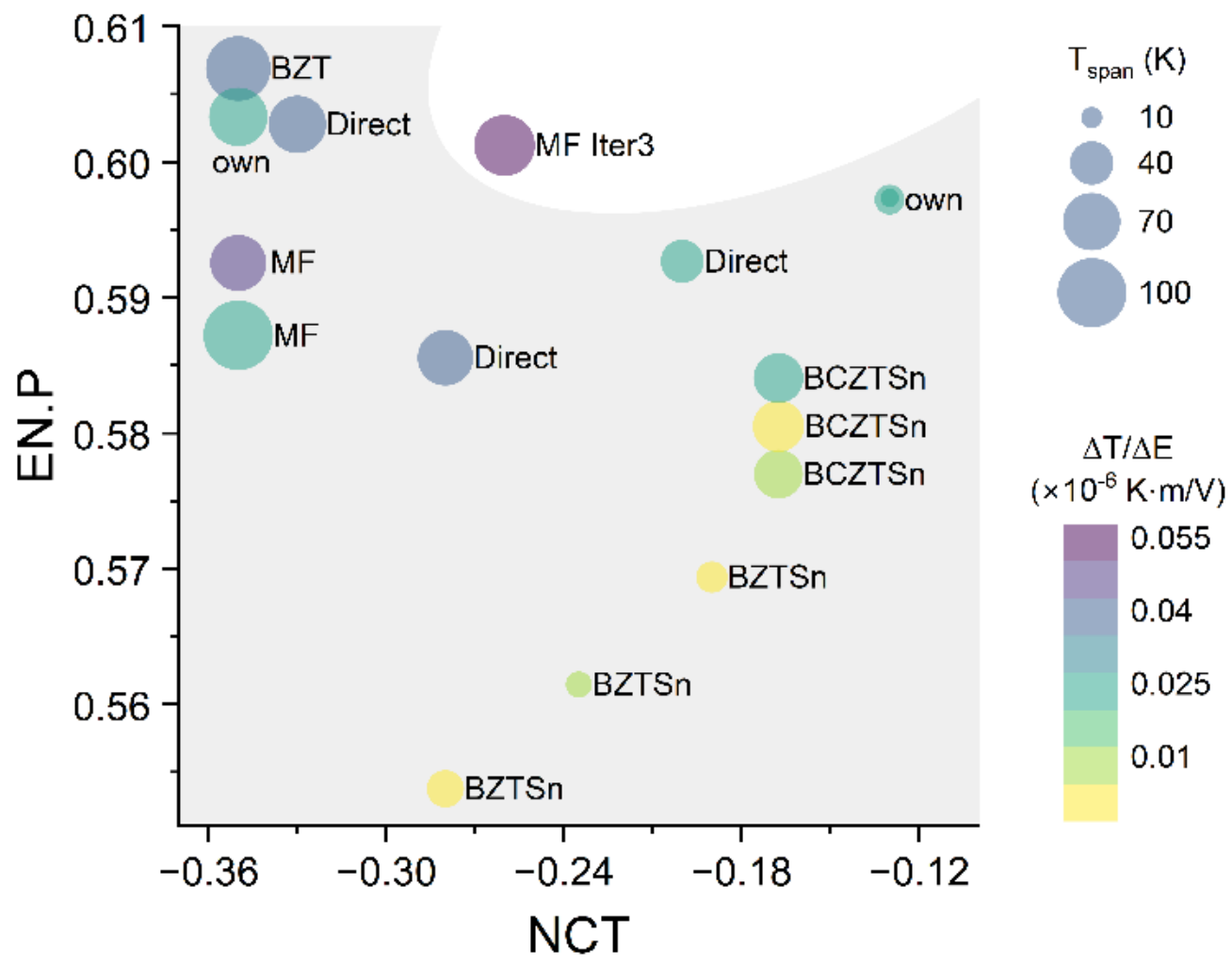

Fig. S8. Feature-space comparison of the optimized composition with literature-reported low-temperature electrocaloric ceramics in the NCT-EN.P descriptor space. Bubble size represents the operational temperature span (T_{span}), and color represents the electrocaloric strength ($\Delta T/\Delta E$) at -70 °C. "Direct" and "MF" denote compositions selected based on direct-only and multi-fidelity models, respectively, while "MF Iter3" represents the final composition identified after the third active-learning iteration.

REFERENCES

[1] M. Kennedy, A. O'Hagan, Predicting the output from a complex computer code when fast approximations are available, Biometrika 87(1) (2000) 1-13.
[2] L. Le Gratiet, Multi-fidelity Gaussian process regression for computer experiments, Université Paris-Diderot Paris, 2013.
[3] A.I.J. Forrester, A. Sóbester, A.J. Keane, Multi-fidelity optimization via surrogate modelling, Proceedings of the Royal Society A: Mathematical, Physical and Engineering Sciences 463(2088) (2007) 3251-3269.
[4] R. Yuan, Z. Liu, P.V. Balachandran, D. Xue, Y. Zhou, X. Ding, J. Sun, D. Xue, T. Lookman, Accelerated Discovery of Large Electrostrains in BaTiO3-Based Piezoelectrics Using Active Learning, Advanced Materials 30(7) (2018) 1702884.
[5] R. Yuan, D. Xue, D. Xue, Y. Zhou, X. Ding, J. Sun, T. Lookman, The Search for BaTiO3-Based Piezoelectrics With Large Piezoelectric Coefficient Using Machine

Learning, IEEE Transactions on Ultrasonics, Ferroelectrics, and Frequency Control 66(2) (2019) 394-401.
[6] R. Yuan, Y. Tian, D. Xue, D. Xue, Y. Zhou, X. Ding, J. Sun, T. Lookman, Accelerated Search for BaTiO3-Based Ceramics with Large Energy Storage at Low Fields Using Machine Learning and Experimental Design, Advanced Science 6(21) (2019) 1901395.
[7] R.D. Shannon, Revised effective ionic radii and systematic studies of interatomic distances in halides and chalcogenides, Acta Crystallographica Section A 32(5) (1976) 751-767.
[8] C.J. Bartel, C. Sutton, B.R. Goldsmith, R. Ouyang, C.B. Musgrave, L.M. Ghiringhelli, M. Scheffler, New tolerance factor to predict the stability of perovskite oxides and halides, Science Advances 5(2) (2019) eaav0693.
[9] C. Li, K.C.K. Soh, P. Wu, Formability of ABO3 perovskites, Journal of Alloys and Compounds 372(1) (2004) 40-48.
[10] N.E. Brese, M. O'Keeffe, Bond-valence parameters for solids, Acta Crystallographica Section B 47(2) (1991) 192-197.
[11] WebElements. https://www.webelements.com.
[12] A. Stolyarenko, DATABASE ON PROPERTIES OF CHEMICAL ELEMENTS, 2020. https://phases.imet-db.ru/elements/main.aspx.
[13] R.G. Pearson, Absolute electronegativity and hardness: application to inorganic chemistry, Inorganic Chemistry 27(4) (1988) 734-740.
[14] J. William D. Callister, D.G. Rethwisch, Materials Science and Engineering: An Introduction (8th-edition), Wiley2010.
[15] K.M. Rabe, J.C. Phillips, P. Villars, I.D. Brown, Global multinary structural chemistry of stable quasicrystals, high-T_C ferroelectrics, and high-T_C superconductors, Physical Review B 45(14) (1992) 7650-7676.
[16] P. Schwerdtfeger, J.K. Nagle, 2018 Table of static dipole polarizabilities of the neutral elements in the periodic table, Molecular Physics 117(9-12) (2019) 1200-1225.
[17] E. Clementi, D.L. Raimondi, W.P. Reinhardt, Atomic Screening Constants from SCF Functions. II. Atoms with 37 to 86 Electrons, The Journal of Chemical Physics 47(4) (1967) 1300-1307.
[18] E. Clementi, D.L. Raimondi, Atomic Screening Constants from SCF Functions, The Journal of Chemical Physics 38(11) (1963) 2686-2689.
[19] A.M. James, M.P. Lord, Macmillan's Chemical and Physical Data, Macmillan, London, UK, 1992.
[20] P. Villars, K. Cenzual, J. Daams, Y. Chen, S. Iwata, Data-driven atomic environment prediction for binaries using the Mendeleev number: Part 1. Composition AB, Journal of Alloys and Compounds 367(1) (2004) 167-175.
[21] X. Wang, J. Zhang, X. Ma, H. Luo, L. Liu, H. Liu, J. Chen, Machine learning assisted composition design of high-entropy Pb-free relaxors with giant energy-storage, Nature Communications 16(1) (2025) 1254.
[22] M. Su, R. Grimes, S. Garg, D. Xue, P.V. Balachandran, Machine-Learning-Enabled Prediction of Adiabatic Temperature Change in Lead-Free BaTiO3-Based Electrocaloric Ceramics, ACS Applied Materials & Interfaces 13(45) (2021) 53475-53484.

[23] Z. Sun, L. Diwu, R. Gao, P. Sun, H. Jing, S. Huang, Y. Tian, Z. Wang, L. Jin, D.Q. Tan, Machine Learning-Driven Ultra-High Energy Storage Performance in Ferro-Superparaelectric Capacitors, Advanced Functional Materials 36(10) (2026) e16412.
[24] A. Semenov, I. Mylnikov, A. Es'kov, A. Anokhin, Y. Pavlova, A. Dedyk, A. Kholkin, Influence of thermal cycling rate and sintering temperature of barium titanate and barium strontium titanate ceramics on their dielectric properties, Ferroelectrics 618(4) (2024) 895-905.
[25] H.-T. Kim, J.-H. Ji, B.S. Kim, J.-H. Koh, Strain related curie temperature and improved piezoelectric properties of lead-free (1-x)Ba(Sn,Ti)O3-x(Ba,Ca)TiO3 ceramics, Ceramics International 46(16, Part A) (2020) 25050-25057.
[26] S. Patel, R. Vaish, Effect of sintering temperature and dwell time on electrocaloric properties of Ba0.85Ca0.075Sr0.075Ti0.90Zr0.10O3 ceramics, Phase Transitions 90(5) (2017) 465-474.
[27] L. Curecheriu, M.T. Buscaglia, V.A. Lukacs, L. Padurariu, C.E. Ciomaga, Role of Density and Grain Size on the Electrocaloric Effect in Ba0.90Ca0.10TiO3 Ceramics, Materials 15(21) (2022) 7825.
[28] W. Xu, X. Huang, H. Li, C. Lin, X. Wu, M. Gao, T. Lin, C. Zhao, Calcination temperature-associated discontinuous grain size effect on electrocaloric properties in fine-/large-grain BaTiO3-based ferroelectric ceramics, Journal of the European Ceramic Society 44(12) (2024) 7071-7080.
[29] D. Xue, D. Xue, R. Yuan, Y. Zhou, P.V. Balachandran, X. Ding, J. Sun, T. Lookman, An informatics approach to transformation temperatures of NiTi-based shape memory alloys, Acta Materialia 125 (2017) 532-541.
[30] Z. Kutnjak, B. Rožič, Indirect and Direct Measurements of the Electrocaloric Effect, in: T. Correia, Q. Zhang (Eds.), Electrocaloric Materials: New Generation of Coolers, Springer Berlin Heidelberg, Berlin, Heidelberg, 2014, pp. 147-182.
[31] B. Rožič, B. Malič, H. Uršič, J. Holc, M. Kosec, B. Neese, Q.M. Zhang, Z. Kutnjak, Direct Measurements of the Giant Electrocaloric Effect in Soft and Solid Ferroelectric Materials, Ferroelectrics 405(1) (2010) 26-31.
[32] C. Molin, J. Peräntie, F. Le Goupil, F. Weyland, M. Sanlialp, N. Stingelin, N. Novak, D.C. Lupascu, S. Gebhardt, Comparison of direct electrocaloric characterization methods exemplified by 0.92 Pb(Mg1/3Nb2/3)O3-0.08 PbTiO3 multilayer ceramics, Journal of the American Ceramic Society 100(7) (2017) 2885-2892.
[33] N. Novak, F. Weyland, G.A.R. Jr, Electrocaloric properties and caloric figure of merit in the ferroelectric solid solution BaZrO3–BaTiO3 (BZT), Journal of the European Ceramic Society 41(2) (2021) 1280-1287.
[34] H. Kaddoussi, A. Lahmar, Y. Gagou, J.L. Dellis, H. Khemakhem, M.E. Marssi, Electro-caloric effect in lead-free ferroelectric Ba1−xCax(Zr0.1Ti0.9)0.925 Sn0.075O3 ceramics, Ceramics International 41(10, Part B) (2015) 15103-15110.
[35] H. Kaddoussi, Y. Gagou, A. Lahmar, B. Allouche, J.L. Dellis, M. Courty, H. Khemakhem, M. El Marssi, Ferroelectric phase changes and electrocaloric effects in Ba(Zr0.1Ti0.9)1−xSnxO3 ceramics solid solution, Journal of Materials Science 51(7) (2016) 3454-3462.

[36] J. Chen, B. Peng, W. Sun, Pure negative electrocaloric effect achieved by SiN/p-GaN composite substrate, Nano Energy 97 (2022) 107195.
[37] T. Yamada, S. Matsuo, T. Kamo, H. Funakubo, M. Yoshino, T. Nagasaki, Experimental study of effect of strain on electrocaloric effect in (001)-epitaxial (Ba,Sr)TiO3 thin films, Japanese Journal of Applied Physics 56(10S) (2017) 10PF15.
[38] J. Peräntie, H.N. Tailor, J. Hagberg, H. Jantunen, Z.-G. Ye, Electrocaloric properties in relaxor ferroelectric (1−x)Pb(Mg1/3Nb2/3)O3–xPbTiO3 system, Journal of Applied Physics 114(17) (2013).
[39] M. Guo, M. Wu, W. Gao, B. Sun, X. Lou, Giant negative electrocaloric effect in antiferroelectric PbZrO3 thin films in an ultra-low temperature range, Journal of Materials Chemistry C 7(3) (2019) 617-621.
[40] W. Geng, D. Zheng, X. Qiao, X. Wang, L. Zhang, K. Bi, J. He, X. Chou, Giant negative electrocaloric effect in antiferroelectric La-modified PbZrO3 thin films within wide temperature range, Journal of Alloys and Compounds 886 (2021) 161192.
[41] V.S. Bondarev, E.A. Mikhaleva, M.V. Gorev, I.N. Flerov, Direct and indirect studies of the electrocaloric effect in single crystalline ferrielectric (NH4)2SO4, Journal of Alloys and Compounds 892 (2022) 162130.
[42] C. Lei, A.A. Bokov, Z.G. Ye, Relaxor Behavior in Ba(Ti0.72Sn0.28)O3 Solid Solution, Ferroelectrics 339(1) (2006) 129-136.
[43] L. Wu**, X. Wang, J.H. Wang, R. Guo, A.S. Bhalla, Ferroelectric Properties and Electric Field Dependent Polarization in 0.86BaTiO3–0.14(Bi3/4Na1/4)(Mg1/4Ti3/4)O3 Ceramics, Ferroelectrics Letters Section 37(1) (2010) 1-9.
[44] Z. Yu, C. Ang, R. Guo, A.S. Bhalla, Ferroelectric-relaxor behavior of Ba(Ti0.7Zr0.3)O3 ceramics, Journal of Applied Physics 92(5) (2002) 2655-2657.
[45] V. Kraft, U.R. Eckstein, M. Kuhfuß, N.H. Khansur, F.P. Marlton, B. Mullens, A. Martin, K. Hayashi, K.G. Webber, Temperature-dependent electromechanical response of $BaTi_{1-x}Sn_xO_3$: Analysis of reversible and irreversible contributions, Journal of the American Ceramic Society 109(1) (2026) e70233.
[46] B. Kaeswurm, F.H. Schader, K.G. Webber, Ferroelectric, ferroelastic, piezoelectric, and dielectric properties of lead zirconate titanate from –150°C to 350°C, Ceramics International 44(2) (2018) 2358-2363.
